# Pardaxin, an Antimicrobial Peptide, Triggers Caspase-Dependent and ROS-Mediated Apoptosis in HT-1080 Cells

**DOI:** 10.3390/md9101995

**Published:** 2011-10-19

**Authors:** Tsui-Chin Huang, Jheng-Fong Lee, Jyh-Yih Chen

**Affiliations:** Marine Research Station, Institute of Cellular and Organismic Biology, Academia Sinica, 23-10 Dahuen Rd., Jiaushi, Ilan 262, Taiwan; E-Mails: tsuichin@gmail.com (T.-C.H.); cinsear@hotmail.com (J.-F.L.)

**Keywords:** antimicrobial peptide, pardaxin, apoptosis

## Abstract

Pardaxin is an antimicrobial peptide (AMP) that was first isolated from secretions of the Red Sea Moses sole. The role of pardaxin in inducing apoptosis for preventing cancer has not yet been investigated. In the present study, we examined the antitumor activity of pardaxin against human fibrosarcoma HT-1080 cells; pardaxin inhibited cell proliferation by inducing apoptosis, as demonstrated by an increase in the externalization of plasma membrane phosphatidylserine and the presence of chromatin condensation. Additionally, pardaxin-treated cells showed elevation of caspase-3/7 activities, disruption of the mitochondrial membrane potential, and accumulation of reactive oxygen species (ROS) production. Inhibition of ROS production and caspase-3/7 activities reduced pardaxin-induced effects. Taken together, these findings suggest that pardaxin may be a potential anticancer agent for selectively inducing apoptosis in cancer cells.

## 1. Introduction

Apoptosis is a fundamental cellular process regulating development that was first described in *Caenorhabditis elegans* in the early 1980s [1]. Genetic studies discovered various protein families in mammals that are homologous to those in *C. elegans*, e.g., Cys proteases, which are involved in carrying out apoptosis [2,3]. This evolutionarily conserved programmed cell death plays a crucial role in determining a cell’s fate and responses to stresses in multicellular organisms [4].

The canonical apoptotic process triggers extrinsic or intrinsic caspase-dependent pathways [5]. In the extrinsic pathway, membrane-bound death receptors face the exterior of plasma membranes to receive stimuli of proapoptotic ligands and transmit signals by activating downstream initiators, caspases-8/10 [5,6]. In the intrinsic pathway, the transduction of signals is due to B-cell lymphoma (BCL)-2 protein family-induced cytochrome (Cyt) c release from mitochondrial intermembrane spaces by proapoptotic stimuli, such as oxidative stress and DNA damage [7–9]. Cytosolic Cyt c recruits apoptotic protease-activating factor (APAF)-1 and procaspase-9 to form apoptosomes [10]. Activated caspases-8, -9, and -10 promote apoptosis by cleaving effector caspases, including caspases-3 and -7, into the active forms which causes proteolysis of their substrates and inhibits the normal physiological functioning of unhealthy cells [11,12].

Apoptotic cells undergo unique changes in their appearance, including cell shrinkage, nuclear fragmentation, chromatin condensation, and membrane blebbing [13]. A deficiency of apoptosis is associated with various diseases, including cancer [14]. Compounds that induce apoptosis in tumor cells are considered potential agents against cancer [14].

Antimicrobial peptides (AMPs) are evolutionarily conserved molecules that provide innate immune responses in a variety of invertebrate, plant, and animal species [15]. AMPs are generally cationic and amphipathic in their amino acid composition [16]. Since most bacterial surfaces are anionic, the peptides can bind to target organisms by electrostatic interactions [17]. AMPs subsequently kill bacteria by disrupting the membrane structure or inhibiting fundamental metabolism [15,18]. In addition to the antibacterial activities of AMPs, recent studies showed selective activities in anticancer effects due to the negatively charged glycosylation characteristics of membrane proteins of cancer cells [19,20].

Pardaxin, a polypeptide composed of 33 amino acid residues in its active form, is an AMP that was first isolated from secretions of the fish, the Red Sea Moses sole [21]. Pardaxin was synthesized and showed the same biological activity as the natural form [22]. With pore-forming ability on lipid membranes [23], pardaxin demonstrated various potential antibacterial, antiviral, and neurotoxic activities [22–25]. Our previous study revealed that pardaxin induced apoptosis in human cervical adenocarcinoma HeLa cells [26]. However, the cytotoxic effects and mechanisms of the anticancer activity of pardaxin in other cancers remain unclear.

In this study, we examined the cytotoxic efficacy and investigated the molecular mechanisms underlying the anticancer activity of synthetic pardaxin in human fibrosarcoma HT-1080 cells. Our results showed that the synthetic pardaxin-induced apoptosis in HT-1080 cells was caspase-dependent and ROS-mediated.

## 2. Results

### 2.1. Pardaxin Inhibited Cell Proliferation in Human Fibrosarcoma HT-1080 Cells

**Figure 1:**
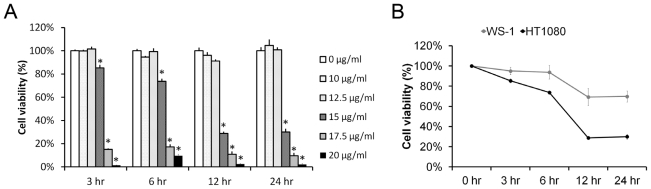
Cell viability of pardaxin-treated HT-1080 and WS1 cells. (**A**) HT-1080 cells were treated with 0, 10, 12.5, 15, 17.5, or 20 μg/mL pardaxin. Cell viability was measured by an MTS assay at 0, 3, 6, 12, and 24 h after treatment. Dosages which caused a significant decrease in cell growth compared to the untreated control at each time point are indicated by an asterisk (*p* < 0.01); (**B**) With 15 μg/mL pardaxin treatment, cell viability was measured by an MTS assay at 0, 3, 6, 12, and 24 h in human HT-1080 fibrosarcoma cells and human WS1 fibroblasts.

To clarify whether pardaxin can inhibit the growth of cancer, we treated HT-1080 cells with pardaxin at concentrations of 0, 10, 12.5, 15, and 20 μg/mL for 3, 6, 12, and 24 h. Cell viability was examined by a formazan-based MTS cell viability assay as described in “Materials and methods”. Results showed time- and dose-dependent inhibition of cell growth by pardaxin (Figure 1A). The 50% inhibitory concentrations (IC_50_) were calculated by fitting the data with a sigmoidal model as follows: 15.74 ± 0.83, 15.40 ± 0.20, 14.51 ± 0.18, and 14.52 ± 0.18 μg/mL at 3, 6, 12, and 24 h, respectively. Furthermore, the data also showed that pardaxin selectively inhibited the growth of HT-1080 cancer cells but with a relatively slight effect on normal WS1 fibroblasts at a concentration of 15 μg/mL for 24 h (Figure 1B).

### 2.2. Pardaxin Induced Apoptosis in HT-1080 Cells

**Figure 2:**
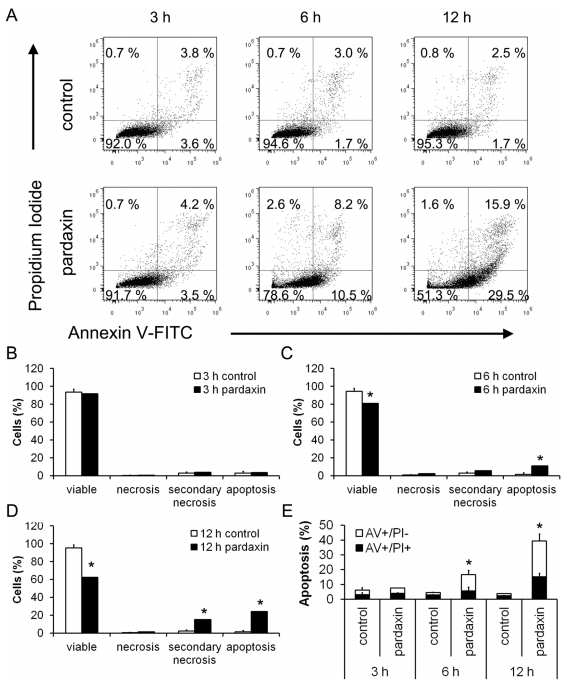
Pardaxin induced apoptotic cell death in HT-1080 cells. HT1080 cells were treated with 15 μg/mL pardaxin. Pardaxin-treated and untreated cells were harvested after 3, 6, and 12 h for annexin V (AV)/propidium iodide (PI) staining, examined by flow cytometry, and analyzed by FlowJo using bi-exponential scaling. (**A**) The x-axis is the annexin V signal which represents the expression of phosphatidylserine on the membrane when cells undergo apoptosis. The y-axis is the PI signal which represents the loss of membrane integrity of cells undergoing necrosis. The lower left, upper left, lower right, and upper right portions respectively indicate viable, necrotic, apoptotic, and secondary necrotic cells. Cells treated with 15 μg/mL pardaxin for (**B**) 3, (**C**) 6, and (**D**) 12 h, were analyzed by flow cytometry in triplicate; (**E**) The sum of apoptotic and secondary necrotic cells was calculated and represents all apoptotic events.

**Figure 3:**
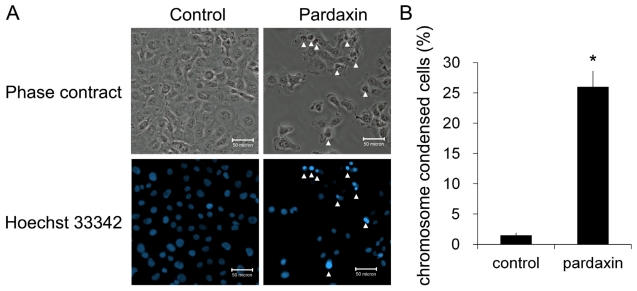
Pardaxin induced apoptosis of HT-1080 cells as examined by nuclear staining. HT-1080 cells were treated with 15 μg/mL pardaxin for 12 h. (**A**) Morphological assessment of apoptosis detected by Hoechst 33342 staining in triplicate. Hoechst staining and phase-contrast images of the same field were observed under fluorescence microscopy. Cells with arrows are apoptotic. Scale bars indicate 50 μm; (**B**) The number of apoptotic cells was calculated and normalized to the number of total cells (*n* = 9, *p* < 0.001).

Since cell viability was significantly inhibited by pardaxin, it was critical to classify which type of cell death was induced in HT-1080 cells. An annexin V/PI assay was performed to detect the type of cell death induced by pardaxin. With 15 μg/mL pardaxin treatment for 12 h, the cell population was distributed in the viable portion to the apoptotic and secondary necrotic portions, and post-apoptotic events (Figure 2A). Apoptotic cells significantly increased from 6 h of treatment, and secondary necrotic cells were subsequently elevated at 12 h (Figure 2B–D). Totals of apoptotic cells (apoptotic and secondary necrotic) significantly increased to 16.67% at 6 h and 39.40% at 12 h (Figure 2E). Pardaxin also increased the number of condensed nuclei from 1.44% to 25.96% (Figure 3A,B). This result indicates that pardaxin induced apoptotic cell death in HT-1080 cells.

### 2.3. Pardaxin Activated Caspase-3/7 Activities

**Figure 4:**
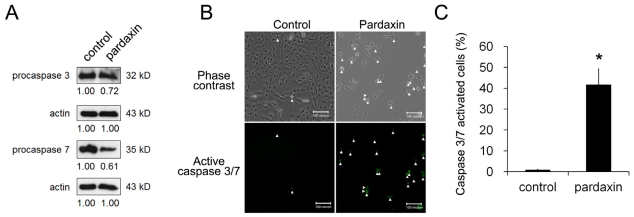
Pardaxin activated caspase-3/7 activities. (**A**) Western blot analysis of intracellular levels of procaspases-3 and -7 was followed by treatment with 15 μg/mL pardaxin for 12 h. Caspase-3/7 activities were assayed by the CellEvent™ caspase-3′7 green detection reagent. Active caspase-3/7 staining and phase-contrast images of the same field were observed under fluorescence microscopy; (**B**) Apoptotic cells are indicated by arrows. Results obtained in a representative experiment are shown. Scale bars indicate 100 μm; (**C**) The mean ± SD of the percentage of apoptotic cells was obtained from three independent experiments. Statistical analyses indicate a significant (*p* < 0.01) increase in cells treated with pardaxin.

We next examined whether caspase activity increased with pardaxin. After 12 h of treatment with 15 μg/mL pardaxin, caspase-3/7 activities were examined by Western blotting or directly assessed by the CellEvent caspase-3/7 green detection reagent which is a nucleic acid-binding dye that harbors the caspase-3/7 cleavage sequence, DEVD, and is fluorescent after being cleaved and bound to DNA. The amount of procaspases-3 and -7 decreased after pardaxin treatment (Figure 4A). On microscopic observations, activated caspase-3/7 signals significantly increased after pardaxin treatment in three independent experiments (Figure 4B,C).

### 2.4. Pardaxin Caused the Loss of the Mitochondrial Membrane Potential (MMP) and the Release of Cyt c in HT-1080 Cells

**Figure 5:**
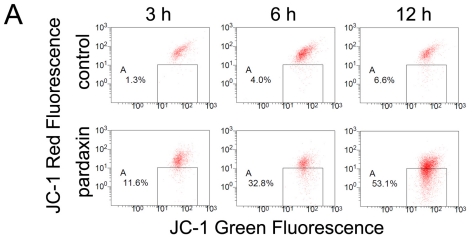

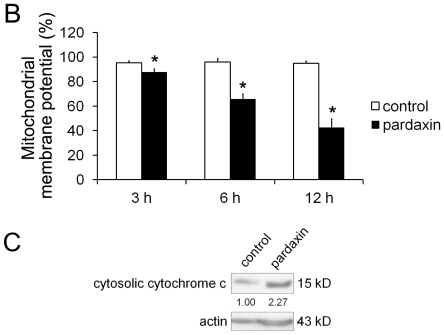
Pardaxin caused loss of the mitochondrial membrane potential (MMP) and release of cytochrome c. HT-1080 cells treated with 15 μg/mL pardaxin for 3, 6, and 12 h were trypsinized to evaluate the MMP by JC-1 monomer/aggregate staining. (**A**) Numbers given in the boxed area represent percentages of cells with depolarized mitochondria. Results obtained in a representative experiment are shown; (**B**) Data are the mean ± SD of the percentage of cells which retained polarized mitochondria from four different experiments. Statistical analyses indicated a significant (*p* < 0.01) decrease in cells with depolarized membranes after pardaxin treatment in a time-dependent manner; (**C**) Western blot analysis of cytosolic cytochrome c was followed by 15 μg/mL pardaxin for 12 h.

Because the loss of MMP acts as a key regulator in the intrinsic apoptosis pathway, we next examined if the MMP was affected by pardaxin. After treatment with 15 μg/mL pardaxin for 3, 6, and 12 h, the population of red^+^/green^+^ cells decreased and shifted to a red^−^/green^+^ population (Figure 5A). MMPs of pardaxin-treated cells respectively decreased to 87.77%, 65.77%, and 42.70% compared to the untreated control at 3, 6, and 12 h of treatment (Figure 5B. Pardaxin also induced the release of Cyt c into the cytosol (Figure 5C) and triggered the intrinsic apoptosis pathway. This suggests that pardaxin might initiate apoptosis through depolarization of the MMP.

### 2.5. Pardaxin Increased the Production of ROS in HT-1080 Cells

**Figure 6:**
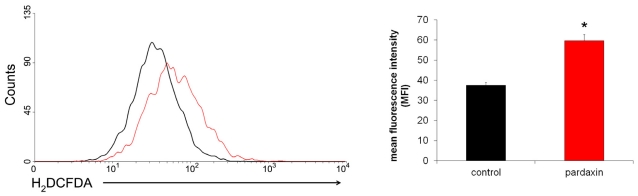
Pardaxin induced production of reactive oxygen species (ROS) in HT-1080 cells. HT-1080 cells treated with 15 μg/mL pardaxin for 4 h were harvested, and baseline ROS production was analyzed by H_2_DCFDA staining. All experiments were performed in triplicate. (**A**) Representative profiles of pardaxin-treated and control cells are illustrated by WinMDI 2.9; (**B**) The mean ± SD of the H_2_DCFDA fluorescent signal was analyzed. An asterisk indicates a significant difference between control and pardaxin-treated cells, *p* < 0.01.

ROS production is implicated in mitochondrion-mediated apoptosis [27]. Therefore, the level of hydrogen peroxide in cells treated with 15 μg/mL pardaxin for 12 h was examined by H_2_DCFDA and flow cytometry. The data showed that pardaxin significantly increased the production of ROS in HT-1080 cells compared to untreated cells (Figure 6). Since losses of the MMP and ROS production are critical steps in the occurrence of apoptosis [28], it can be inferred that pardaxin-induced apoptosis might have been due to activation of an intrinsic ROS-dependent mitochondrial pathway.

### 2.6. Pardaxin Activation of Caspases-3/7 Is ROS Mediated

**Figure 7:**
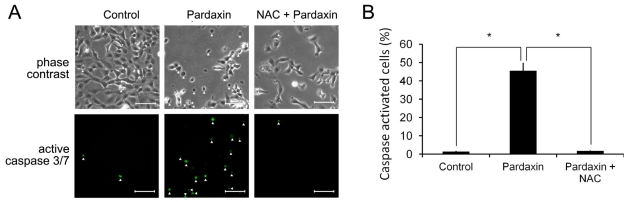
Pardaxin-induced caspase-3/7 activities are ROS mediated. HT-1080 cells were pretreated with 5 μM of the antioxidant, *N*-acetyl-cysteine (NAC), treated with 15 μg/mL pardaxin, or untreated (control) for 12 h. The active caspase-3/7 signal was assayed by the CellEvent™ caspase-3′7 green detection reagent and monitored by fluorescence microscopy. The mean ± SD of the percentage of caspase-activated cells was obtained from three independent experiments. Asterisks indicate a significant (*p* < 0.01) difference in caspase activity compared to the indicated cells. Scale bar is equal to 50 μm.

To examine if pardaxin-triggered caspase activity depends on ROS accumulation, we treated HT-1080 cells with pardaxin in the absence and presence of the antioxidant, *N*-acetyl-cysteine (NAC), an ROS scavenger. Figure 7A shows observations of cell morphology, and the activated caspase-3/7 signals are indicated by white arrows. The morphology of cells subjected to pardaxin plus NAC treatment remained flat and attached which differed from pardaxin-treated cells with had a rounded morphology. Activation of caspases-3/7 was elevated by pardaxin (Figures 4C,7B). Inhibition of ROS production by NAC showed a marked decrease in caspase-3/7 activation after 15 μg/mL pardaxin treatment for 12 h (Figure 7B). It can be inferred that activation of caspases-3/7 was mediated by ROS production.

### 2.7. Pardaxin-Induced Cell Death Is Caspase-3/7 Dependent

**Figure 8:**
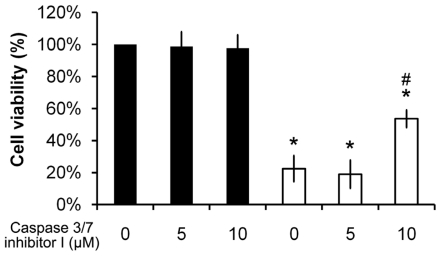
Pardaxin activated caspase-3/7-dependent apoptosis. HT-1080 cells were pretreated with the specific caspase-3/7 inhibitor I at the indicated concentration for 1 h. Cells were then treated with 15 μg/mL pardaxin for 12 h (open column) or left untreated as the control (closed column). Cell viability was measured by an MTS assay. * Indicates a significant (*p* < 0.01) decrease in cell survival compared to control cells (column 1). ^#^ indicates a significant (*p* < 0.01) increase in cell survival compared to pardaxin-treated cells without pretreatment with the caspase inhibitor (column 4).

To clarify whether pardaxin-induced cell death is dependent on caspases-3/7, a caspase-3/7 inhibitor was used. Cell viability was not affected in the presence of 5 or 10 μM of the caspase-3/7 inhibitor I compared to control cells (Figure 8, columns 1–3). Significantly, cell viability of 15 μg/mL pardaxin-treated cells with 10 μM of the caspase-3/7 inhibitor I pretreatment (Figure 8, column 6) recovered to >2-fold that with pardaxin treatment only (Figure 8, column 4). Results suggest that pardaxin-induced cell death is dependent on the activation of caspases-3/7.

## 3. Discussion

Natural and synthetic AMPs exhibit broad activities, including antibacterial, antifungal, antiviral, and anticancer functions [15]. AMPs are a class of peptides composed of cationic residues which range in size from five to 40 amino acids. In some cases, AMPs contain disulfide bond-forming cysteines in their sequences [29]. The stereo properties of AMPs allow them to potently interact with negatively charged substances, for instance bacterial membranes with lipids such as phosphatidylglycerol, cardiolipin, and phosphatidylserine (PS), and the outer membrane anionic lipopolysaccharides of gram-negative bacteria [30]. Plasma membranes of cancer cells are also composed of anionic PS [19] and *O*-glycosylated proteins [20]. On the contrary, AMPs do not electrostatically interact with non-transformed cells, the plasma membranes of which are composed of zwitterionic membrane components, such as sphingomyelin, phosphatidylethanolamine, and phosphatidylcholine [31]. Based on these physical properties of the lipid composition, AMPs thus selectively kill bacterial and malignant cells.

In this study, we investigated the anticancer activity of pardaxin and attempted to provide a molecular explanation of its actions. Pardaxin showed selective activity against HT-1080 cells but not normal WS-1 cells (Figure 1). The selective killing of cancer cells might be attributed to the nature of pardaxin’s cationic sequence which more easily interacts with anionic plasma membranes of cancer cells. There are two major consequences after an AMP binds to a lipid. One is its insertion into the plasma membrane and formation of channel-like pores that cause cell lysis. The other one is its penetration into cells and perturbation of cellular processes.

Herein, we postulated that the uptake of pardaxin into the cytosol may bind to mitochondria which mostly contain the phospholipids, phosphatidylethanolamine, phosphatidic acid, and cardiolipin [32]. This indicates that the MMP was diminished and Cyt c was released into the cytosol of HT-1080 cells after pardaxin treatment (Figure 5), which might have been due to the pore-forming ability of intracellular pardaxin in mitochondrial membranes [33]. The release of Cyt c from mitochondria into the cytosol exhibited a signature of a mitochondrion-mediated apoptotic pathway which was observed in this study (Figures 2 and 3). Subsequently, it in turn triggered activation of caspases 3/7 (Figure 4).

On the other hand, treatment with pardaxin increased ROS production (Figure 6) [34,35], followed by triggering mitochondrion-dependent apoptosis by activating caspase 3/7 activities (Figure 7). The ROS scavenger, NAC, replenishes intracellular levels of the natural antioxidant, glutathione. Although glutathione can serve as an electron donor and tends to reduce disulphide bonds into cysteines, it does not affect the structure of pardaxin which contains no cysteine in its sequence. By scavenging free radicals, NAC can protect cells from ROS-mediated cell death [36]. It was found that depletion of glutathione can lead to accumulation of ROS, decreases in the MMP, release of Cyt c, and activation of caspase 3 and DNA fragmentation, signatures of mitochondria-mediated apoptosis [37].

Chelating of ROS by NAC inhibited pardaxin-triggered caspase 3/7 activation (Figure 7), indicating that caspases act as key regulators in pardaxin-induced apoptosis. Inhibition of caspases 3/7 by a specific inhibitor prevented pardaxin-induced cell death (Figure 8), implying that pardaxin-induced apoptosis is dependent on caspase activity. However, pardaxin-induced cell death only recovered by about 60% in the presence of a caspase 3/7 inhibitor (Figure 8), suggesting that there might be other caspase-independent pathways involved in the mechanism of pardaxin’s action.

## 4. Experimental Section

### 4.1. Materials

Pardaxin (H-GFFALIPKIISSPLFKTLLSAVGSALSSSGGQE-OH) was synthesized and purified to a grade of >95% by GL Biochemistry (Shanghai, China). Synthetic peptides were dissolved in sterile deionized water for the experiments. Minimum essential medium (MEM), fetal bovine serum (FBS), trypsin-EDTA, phosphate-buffered saline (PBS), an annexin V-FITC apoptosis detection kit, MitoProbe JC-1 assay kit, and carboxy-H2DCFDA were purchased from Life Technologies (Carlsbad, CA, USA). Phenazine methosulfate and 3-(4,5-dimethylthiazol-2-yl)-5-(3-carboxymethoxyphenyl)-2- (4-sulfophenyl)-2H-tetrazolium (MTS) were purchased from Promega (Madison, WI, USA). A protein assay kit was purchased from Bio-Rad (Hercules, CA, USA). Rabbit polyclonal antibodies against caspase-3, caspase-7, Cyt c, and horseradish peroxidase (HRP)-conjugated secondary antibodies were purchased from Cell Signaling (Beverly, MA, USA).

### 4.2. Cell Culture

HT-1080 (human fibrosarcoma) cells and WS1 (human skin fibroblast) cells were purchased from the Bioresource Collection and Research Center (BCRC, Taiwan) and cultured at 37 °C in a humidified atmosphere of 5% CO_2_ and 95% air in MEM (Eagle’s medium) supplemented with 2 mM l-glutamine, 0.1 mM non-essential amino acids, and 10% heat-inactivated FBS. All experiments were performed using cells in the logarithmic growth phase.

### 4.3. Cell Viability Assay

We performed an MTS assay to assess cell viability. MTS, a tetrazolium compound, is chemically reduced by cells into formazan, which is soluble in tissue culture medium. Cells were plated in 96-well microplates at an initial density of 2500 cells/well and treated with various concentrations of pardaxin for 3, 6, 12, or 24 h. After treatment, 20 μL of a combined MTS/PMS (phenazine methosulfate) solution (1:1) was added to each well for an additional 2 h. The optical density was measured at 490 nm using a microplate reader. All experiments were performed in triplicate and repeated three times.

### 4.4. Annexin V/Propidium Iodide (PI) Staining

After treatment with or without pardaxin, cells were harvested and washed twice in cold PBS, and resuspended in annexin V-FITC and PI for 30 min in the dark. Cells were measured with a Cytomics FC 500 flow cytometer equipped with an air-cooled argon laser that emitted at 488 nm. Data from at least 10^4^ cells were analyzed with FlowJo software.

### 4.5. Hoechst 33342 Staining

Cells were washed with PBS after incubation in the absence or presence of pardaxin and then incubated in 4% paraformaldehyde for 15 min at 37 °C. After fixation, cells were washed and stained with Hoechst 33342 (1 μg/mL in PBS) for 15 min at 37 °C in the dark. Stained cells were observed under a fluorescence microscope.

### 4.6. Mitochondrial Membrane Potential (MMP) Detection

After treatment with or without pardaxin, cells were harvested and washed twice in cold PBS, and incubated with 2 μm JC-1 for 30 min at 37 °C in the dark. Cells were washed with PBS and resuspended in 500 μL PBS. Stained cells were analyzed on a Cytomics FC 500 flow cytometer to detect green fluorescence at excitation/emission wavelengths of 485/530 nm and red fluorescence at excitation/emission wavelengths of 550/595 nm, respectively.

### 4.7. Measurement of Intracellular ROS

Cells were harvested after incubation in the absence or presence of pardaxin, washed twice in cold PBS, and incubated with 10 μM H_2_DCFDA for 30 min at 37 °C in the dark. Stained cells were analyzed with a Cytomics FC 500 flow cytometer. The mean fluorescence intensity was obtained by histogram statistics using WinMDI 2.9.

### 4.8. Caspase-3/7 Activity Assay

After treatment with or without pardaxin, cells were labeled with 5 μM CellEvent™ caspase-3/7 green detection reagent in complete medium for 30 min at 37 °C in the dark. Stained cells were observed under fluorescence microscopy.

### 4.9. Western Blot Analysis

Cells were harvested, washed twice with ice-cold PBS, and then lysed in lysis buffer containing the protease inhibitor cocktail. The concentration of extracted proteins was determined using the Bio-Rad protein assay reagent. Protein samples were separated by sodium dodecylsulfate polyacrylamide gel electrophoresis (SDS-PAGE) and transferred onto polyvinylidene difluoride membranes. Proteins were detected using polyclonal antibodies and visualized with HRP-conjugated second antibodies under chemiluminescence detection.

### 4.10. Statistical Analysis

Data are expressed as the mean ± standard deviation (SD). Statistical comparisons were performed using Student’s *t*-test, and differences between groups were considered significant at a *p* value of <0.05.

## 5. Conclusions

Chemotherapy is applied as first-line treatment for cancer therapy. Platinum-based chemotherapy causes severe toxicity to cancer cells and also healthy cells with a rapid replication rate as a result of its ability to disrupt DNA repair [38]. The need for novel anticancer drugs with higher specificity targeting malignant tissues is increasingly urgent. In the present study, we show that pardaxin selectively induced apoptosis through an intrinsic apoptotic pathway in cancer cells, thus showing its potential to serve as an anticancer drug. To sum up, our study provides molecular insights into pardaxin-induced cell death in human fibrosarcoma HT-1080 cells. We showed that pardaxin disrupted mitochondrial function and caused an accumulation of ROS that activated a caspase-dependent intrinsic apoptotic pathway. These findings suggest that AMPs can be used as peptide drugs which are target-specific, cost-effective, and easily designed for cancer therapy.

## Supplementary Material
