# Supplementary material for: Pardaxin, an Antimicrobial Peptide, Triggers Caspase-Dependent and ROS-Mediated Apoptosis in HT-1080 Cells
Source: Mar Drugs. 2011 Oct 19;9(10):1995–2009. doi: 10.3390/md9101995 (PMC3210615; doi:10.3390/md9101995)
Supplement: Supplementary file 1 [file marinedrugs-09-01955-s001.pdf]

Supporting Information

Bioactive Cembranoids from the Soft Coral *Sinularia crassa*

Chih-Hua Chao [1,2], Kuei-Ju Chou [1], Chiung-Yao Huang [1], Zhi-Hong Wen [1,3], Chi-Hsin Hsu [1,3], Yang-Chang Wu [4], Chang-Feng Dai [5], and Jyh-Horng Sheu [1,3,*]

[1] Department of Marine Biotechnology and Resources, National Sun Yat-Sen University, Kaohsiung 804, Taiwan; E-Mails: chaochihhua@hotmail.com (C.-H.C.); jzusmile@hotmail.com (K.-J.C.); betty8575@yahoo.com.tw (C.-Y.H.)

[2] Chinese Medicinal Research and Development Center, China Medical University and Hospital, Taichung 404, Taiwan, ROC

[3] Asian Pacific Ocean Research Center, National Sun Yat-sen University, Kaohsiung 804, Taiwan

E-Mails: wzh@mail.nsysu.edu.tw (Z.-H.W.); hsuch@mail.nsysu.edu.tw (C.-H.H.)

[4] College of Chinese Medicine, China Medical University, Taichung 404, Taiwan

E-Mail: yachwu@mail.cmu.edu.tw (Y.-C.W.)

[5] Institute of Oceanography, National Taiwan University, Taipei, Taiwan;

E-Mail: corallab@ntu.edu.tw (C.-F.D.)

For compound 1:

S1-1. ^{1}H NMR spectrum (400 MHz) of compound **1** in $CDCl_3$.

S1-2. ^{13}C NMR spectrum (100 MHz) of compound **1** in $CDCl_3$.

S1-3. ^{1}H NMR spectrum (400 MHz) of compound **1** in C_5D_5N.

S1-4. ^{13}C NMR spectrum (400 MHz) of compound **1** in C_5D_5N.

For compound 2:

S2-1. ^{1}H NMR spectrum (500 MHz) of compound **2** in $CDCl_3$.

S2-2. ^{13}C NMR spectrum (125 MHz) of compound **2** in $CDCl_3$.

S2-3. ^{1}H NMR spectrum (300 MHz) of compound **2** in $CDCl_3$.

S2-4. ^{1}H NMR spectrum (300 MHz) of acetate **1a** in $CDCl_3$.

For compound 3:

S3-1. ^{1}H NMR spectrum (400 MHz) of compound **3** in $CDCl_3$.

S3-2. ^{13}C NMR spectrum (100 MHz) of compound **3** in $CDCl_3$.

For compound 4:

S4-1. ^{1}H NMR spectrum (400 MHz) of compound **4** in $CDCl_3$.

S4-2. ^{13}C NMR spectrum (100 MHz) of compound **4** in $CDCl_3$.

For compound 5:

S5-1. ^{1}H NMR spectrum (400 MHz) of compound **5** in $CDCl_3$.

S5-2. ^{13}C NMR spectrum (100 MHz) of compound **5** in $CDCl_3$.

For compound 6:

S6-1. ^{1}H NMR spectrum (400 MHz) of compound **6** in C_6D_6.

S6-2. ^{13}C NMR spectrum (100 MHz) of compound **6** in C_6D_6.

For compound 7:

S7-1. ^{1}H NMR spectrum (400 MHz) of compound **7** in C_6D_6.

S7-2. ^{13}C NMR spectrum (100 MHz) of compound **7** in C_6D_6.

For compound 8:

S8-1. ^{1}H NMR spectrum (400 MHz) of compound **8** in C_6D_6.

S8-2. ^{13}C NMR spectrum (100 MHz) of compound **8** in C_6D_6.

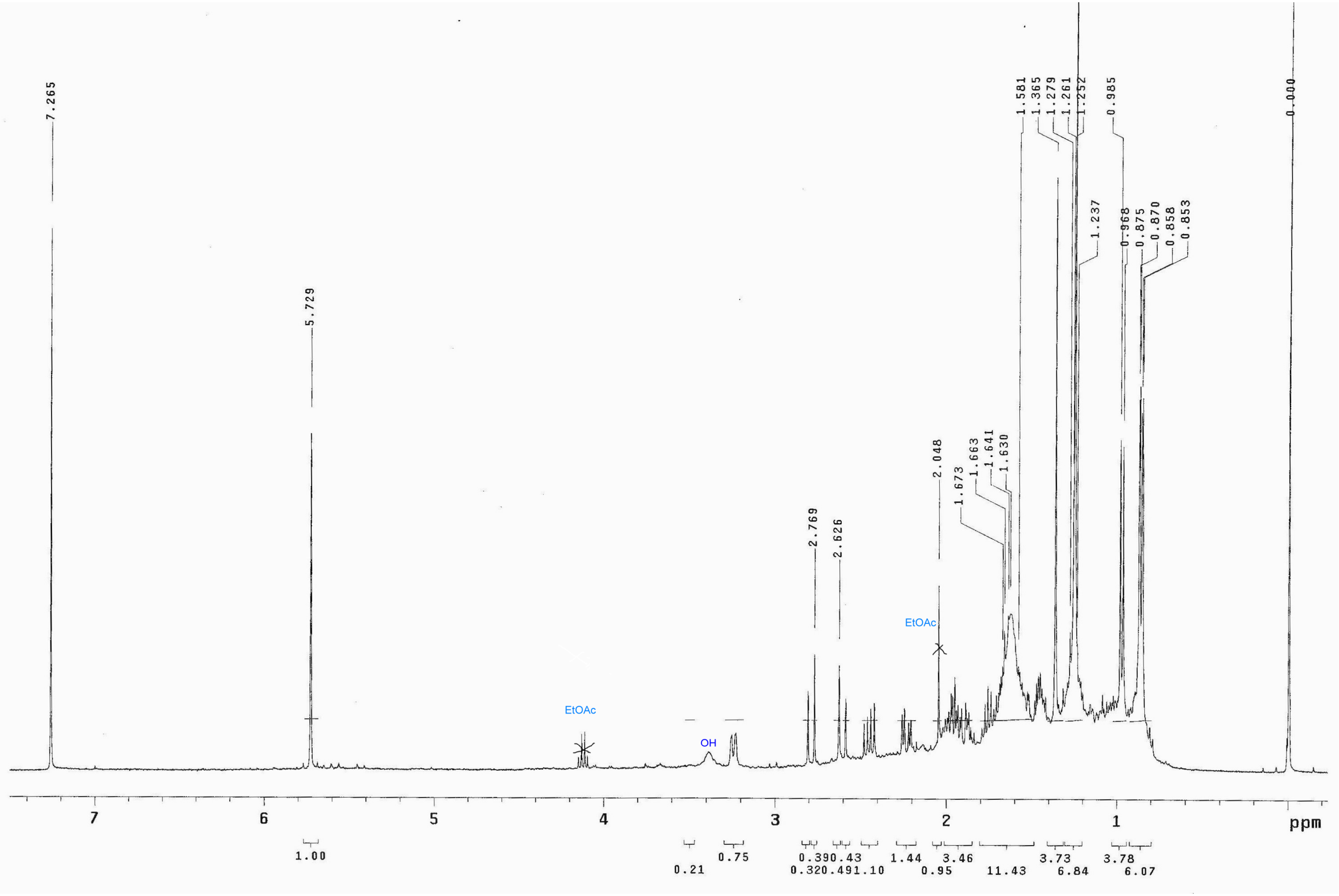

S1-1. ^{1}H NMR spectrum (400 MHz) of compound **1** in $CDCl_3$.

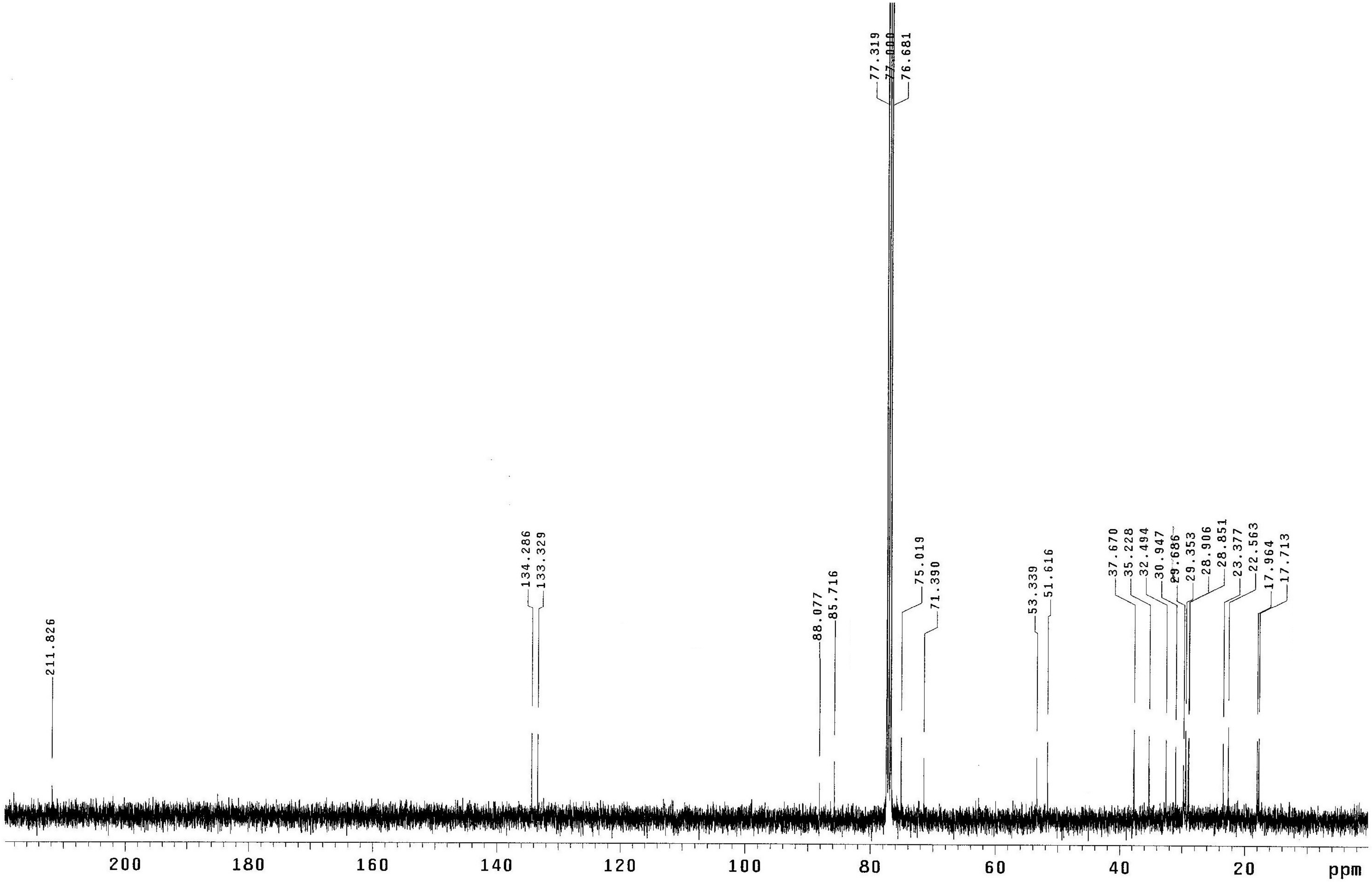

S1-2. ^{13}C NMR spectrum (400 MHz) of compound **1** in $CDCl_3$.

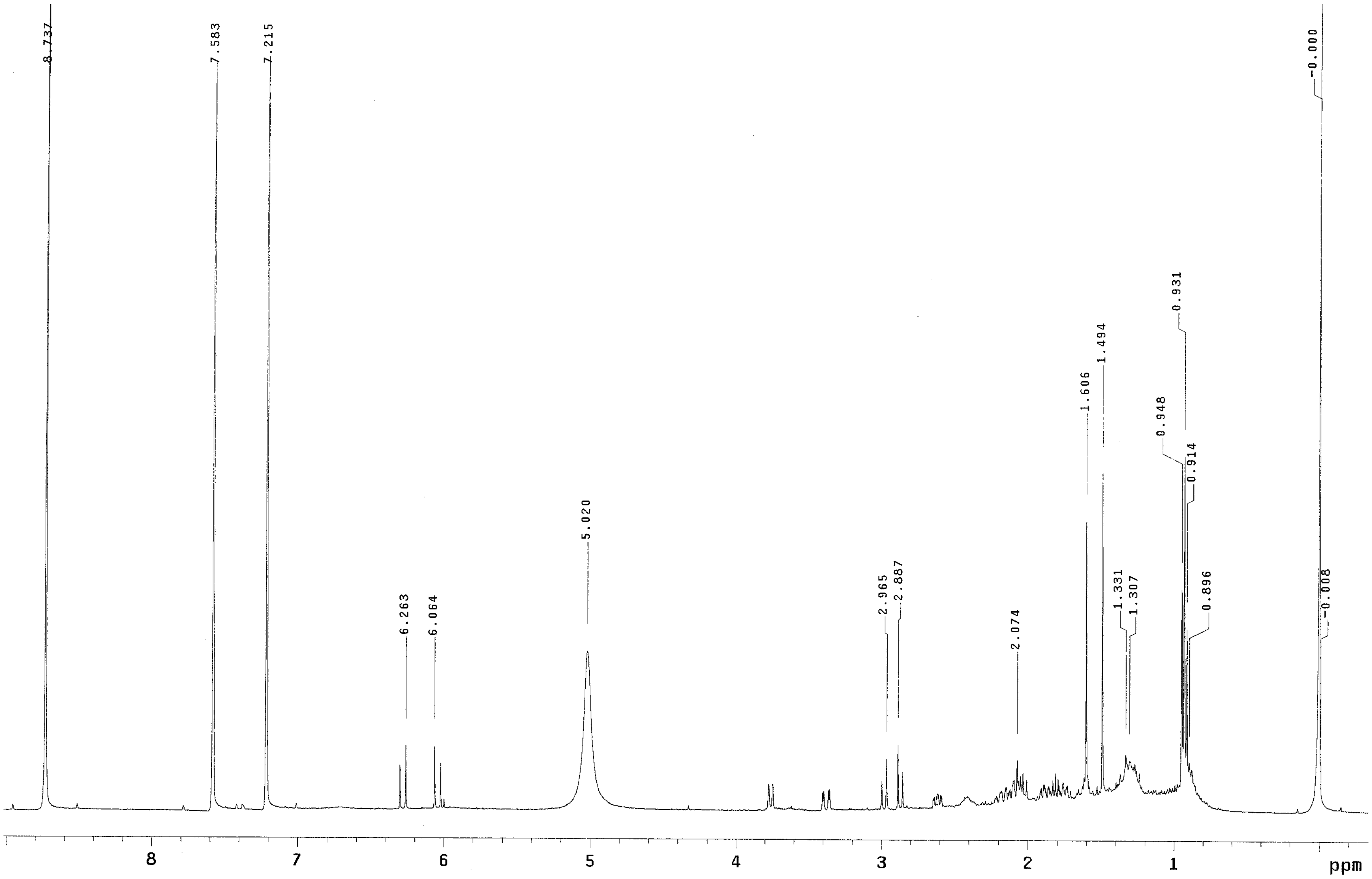

S1-3. ¹H NMR spectrum (400 MHz) of compound **1** in C_5D_5N.

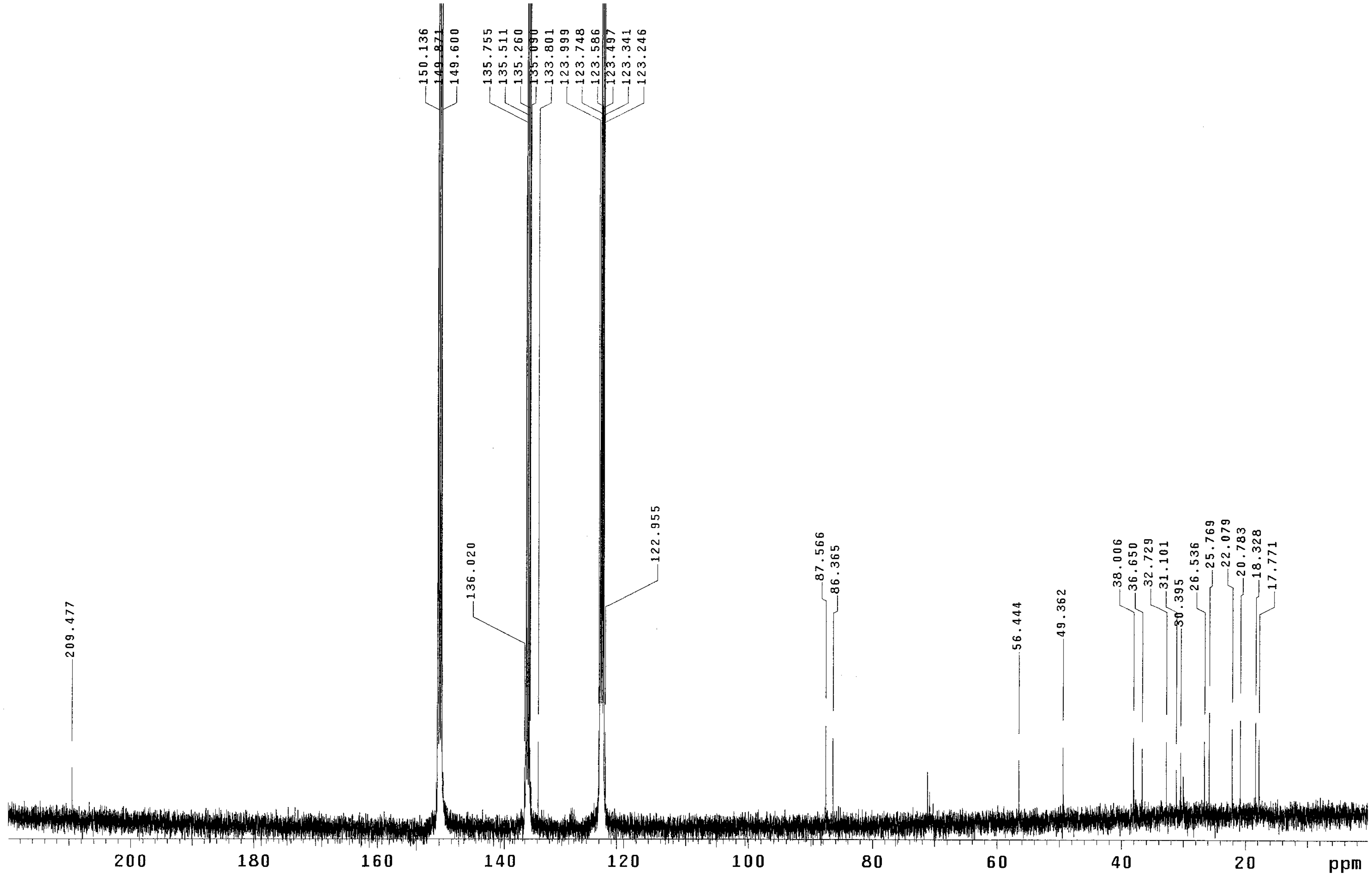

S1-4. ^{13}C NMR spectrum (400 MHz) of compound **1** in C_5D_5N.

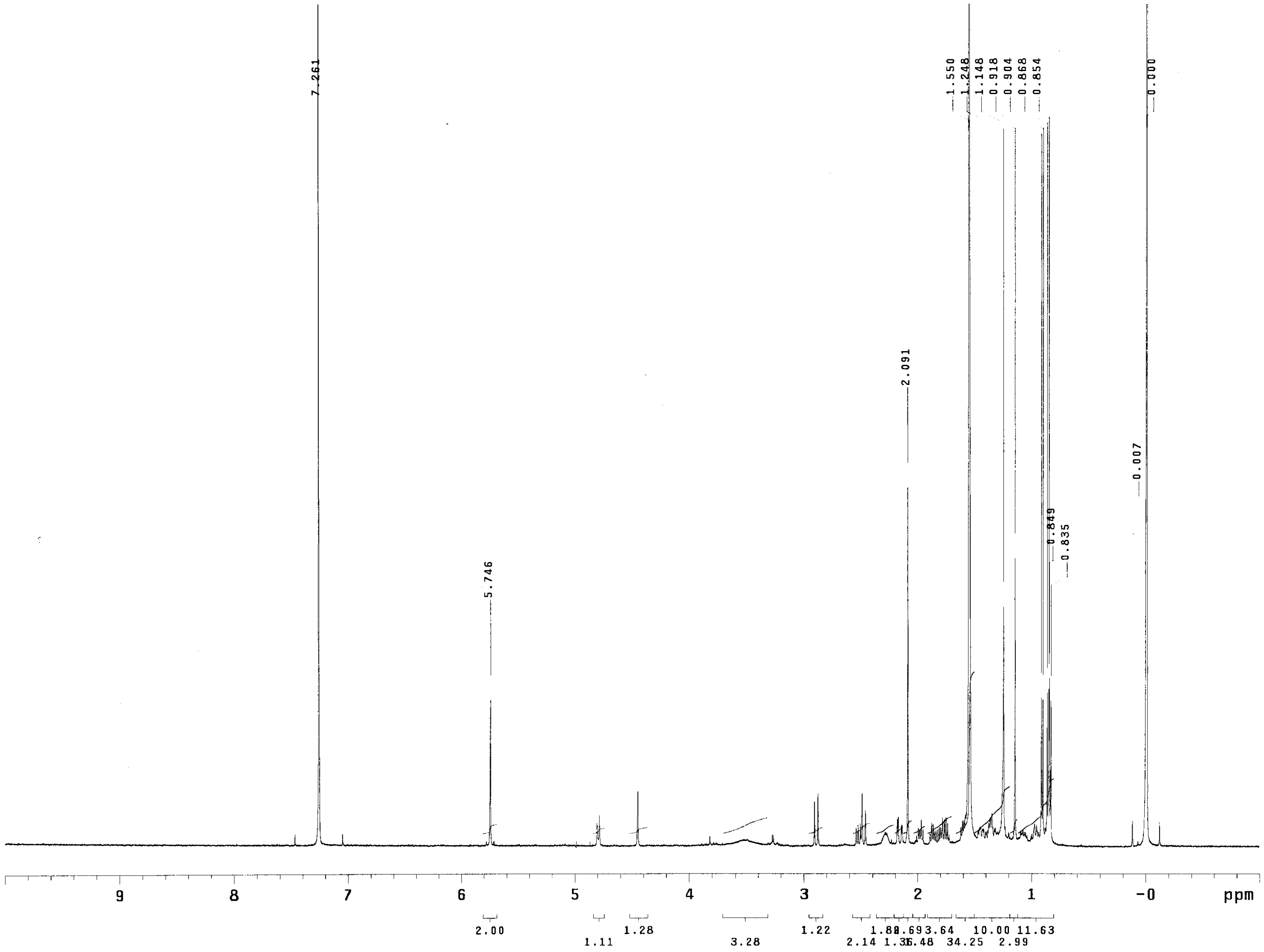

S2-1. ^{1}H NMR spectrum (500 MHz) of compound **2** in $CDCl_3$.

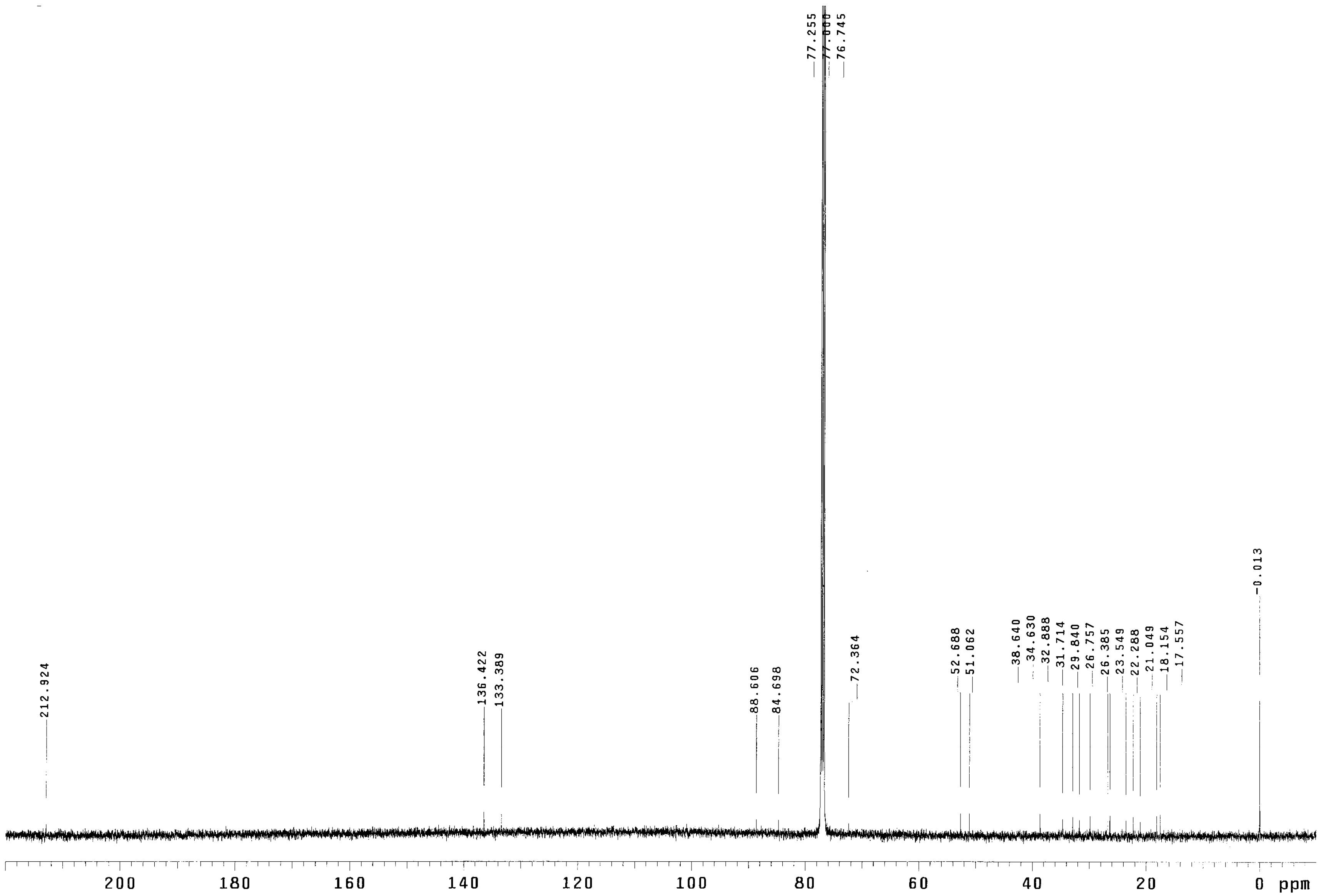

S2-2. ^{13}C NMR spectrum (500 MHz) of compound **2** in $CDCl_3$.

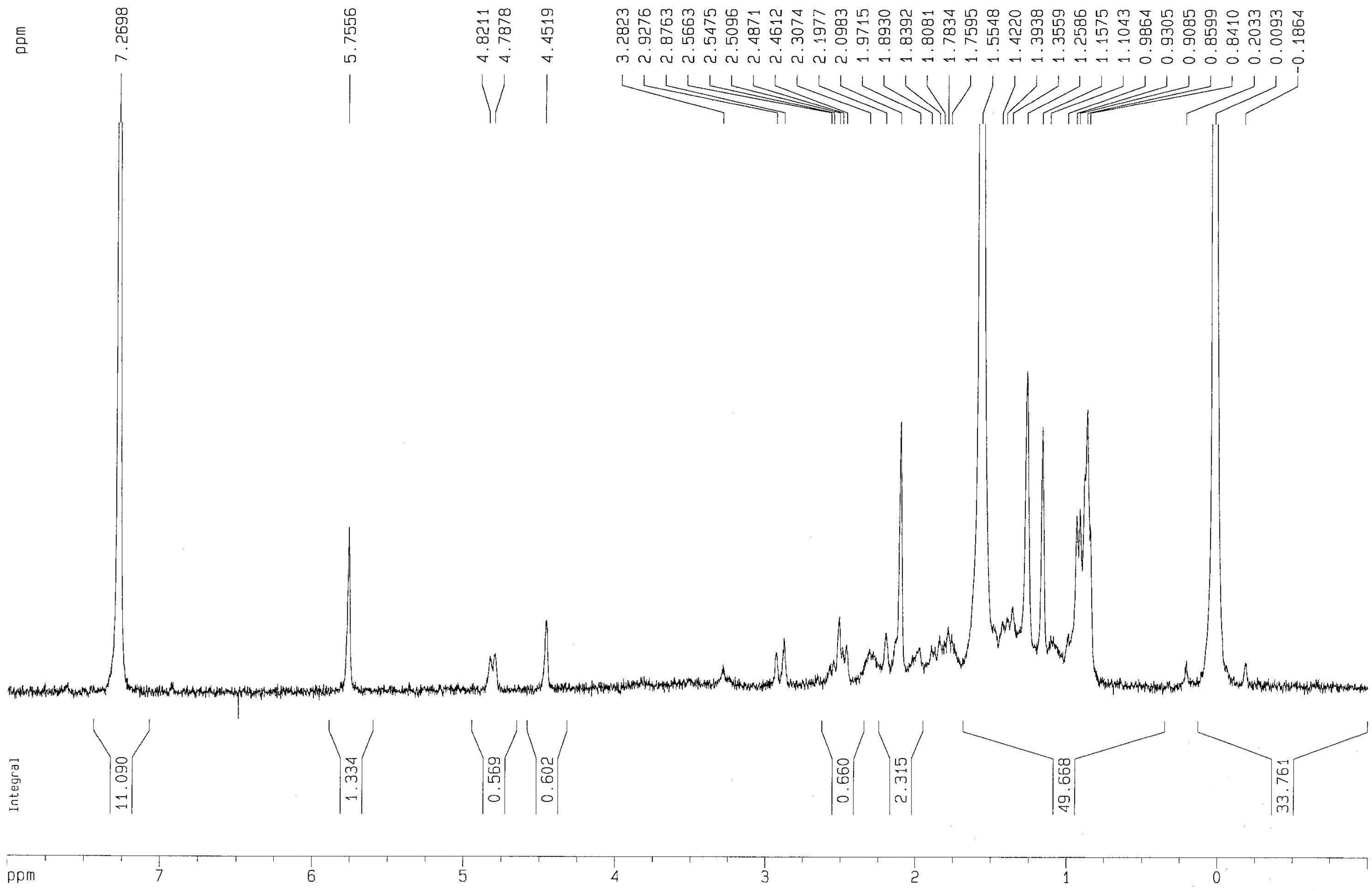

S2-3. ^{1}H NMR spectrum (300 MHz) of compound **2** in $CDCl_3$.

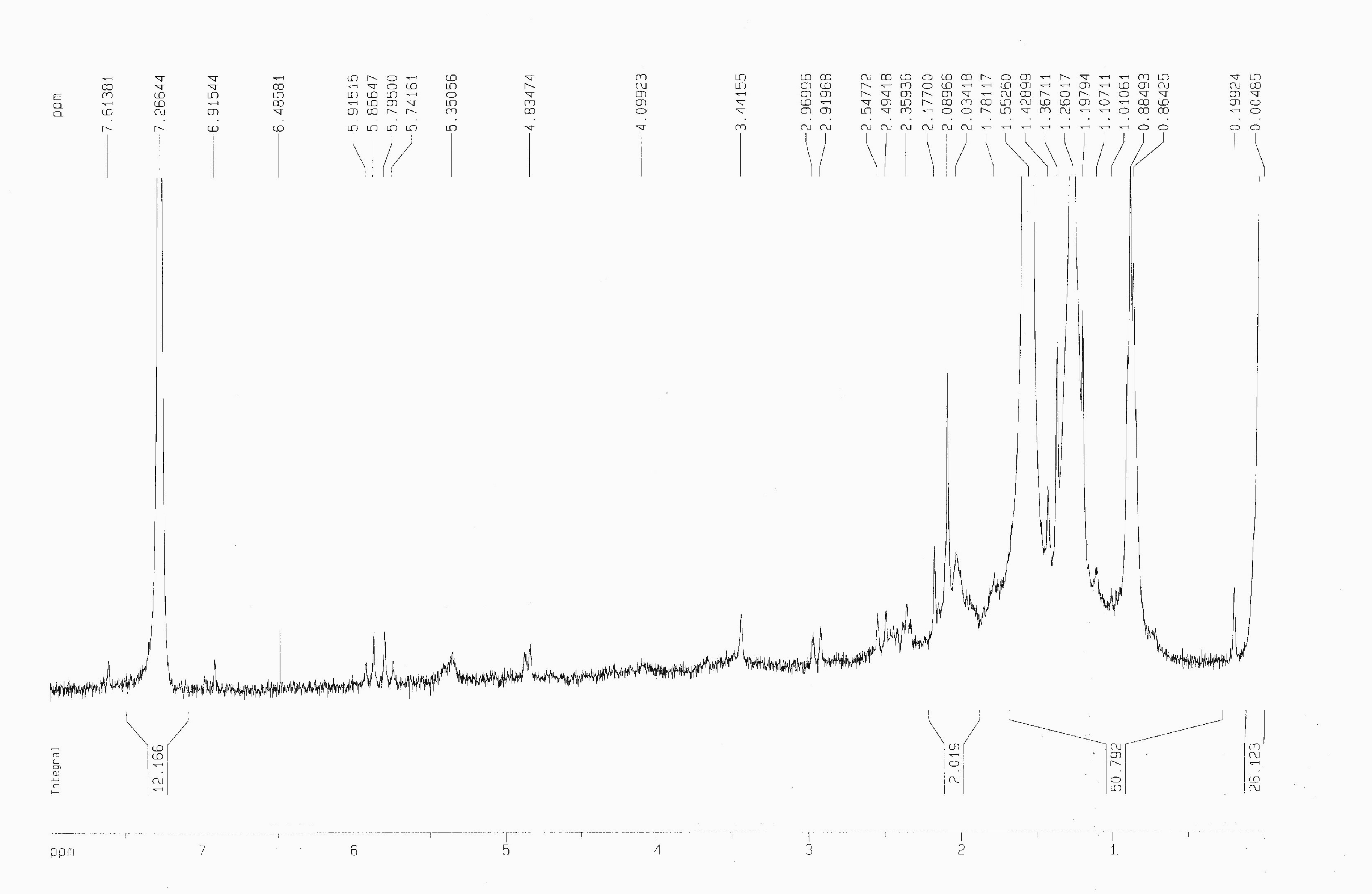

S2-4. ^{1}H NMR spectrum (300 MHz) of acetate **1a** in $CDCl_3$.

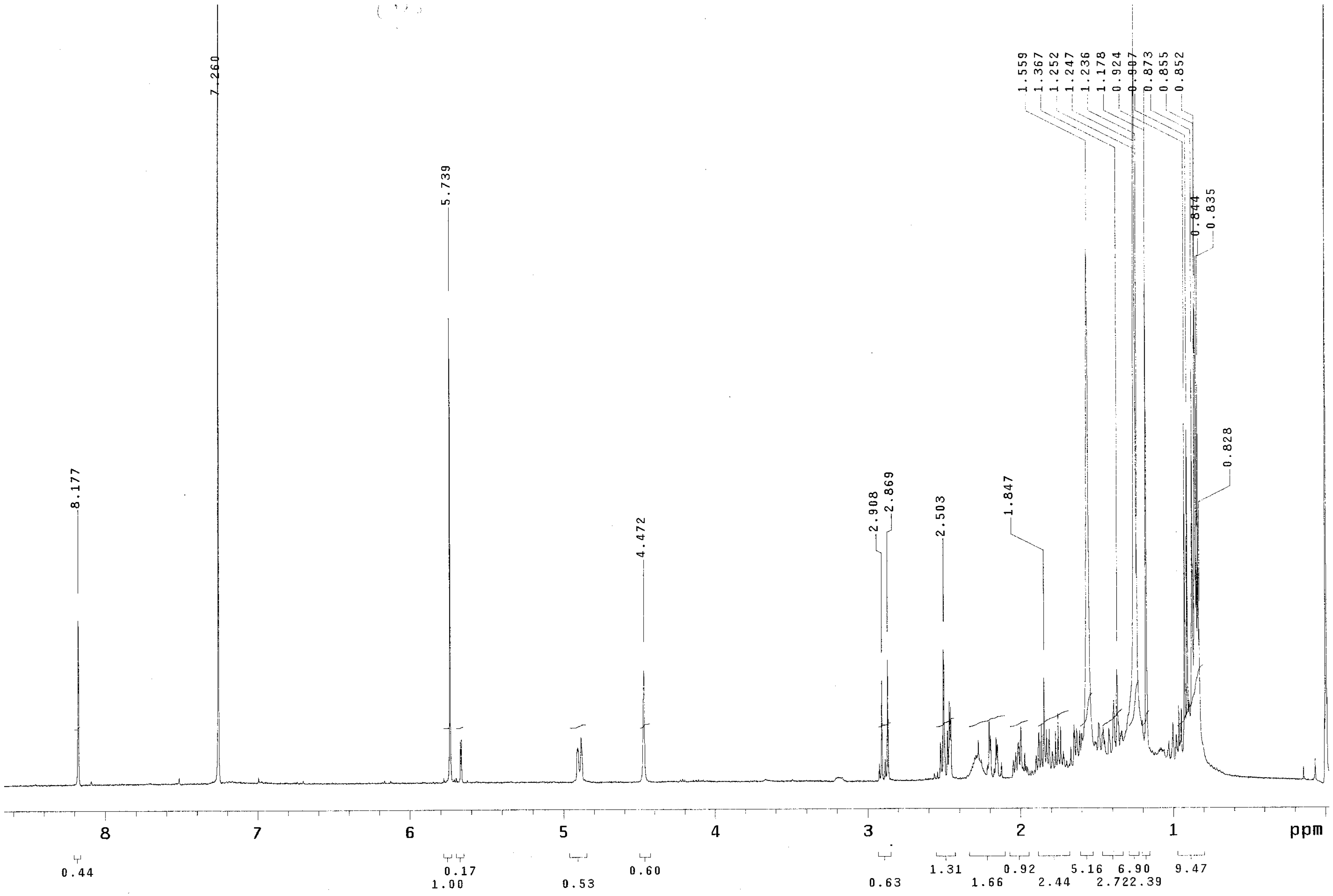

S3-1. ^{1}H NMR spectrum (400 MHz) of compound **3** in $CDCl_3$.

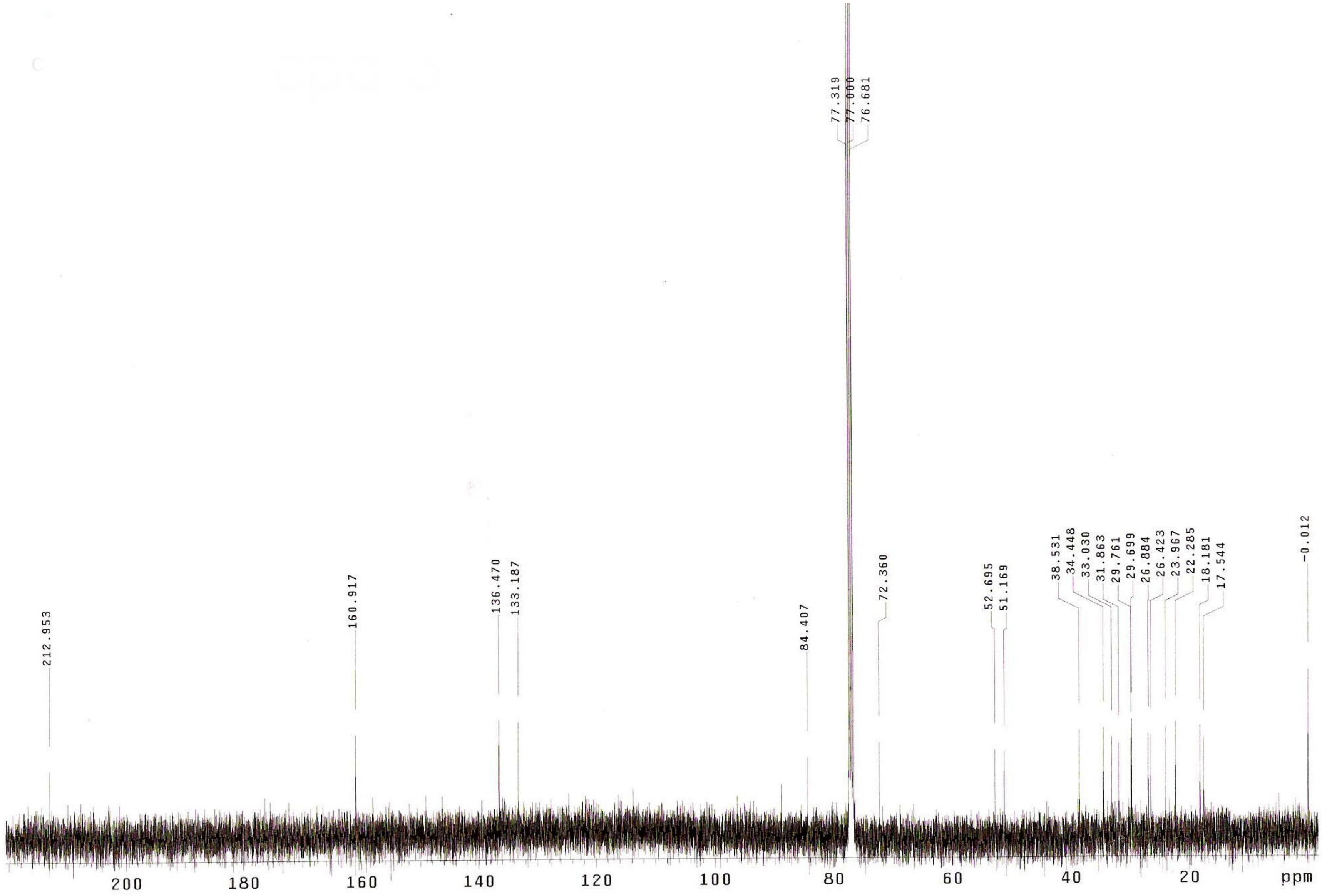

S3-2. ^{13}C NMR spectrum (400 MHz) of compound **3** in $CDCl_3$.

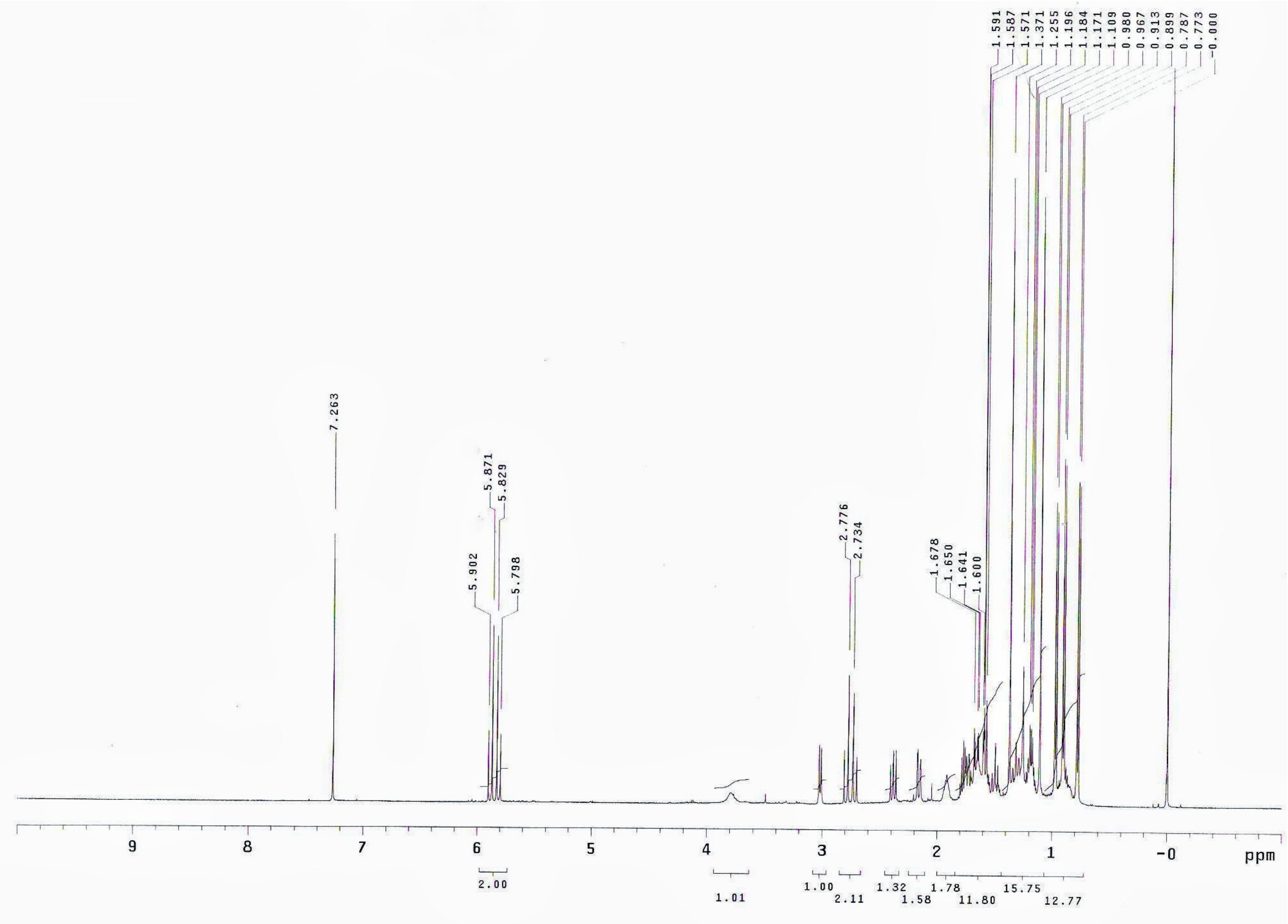

S4-1. ^{1}H NMR spectrum (400 MHz) of compound **4** in $CDCl_3$.

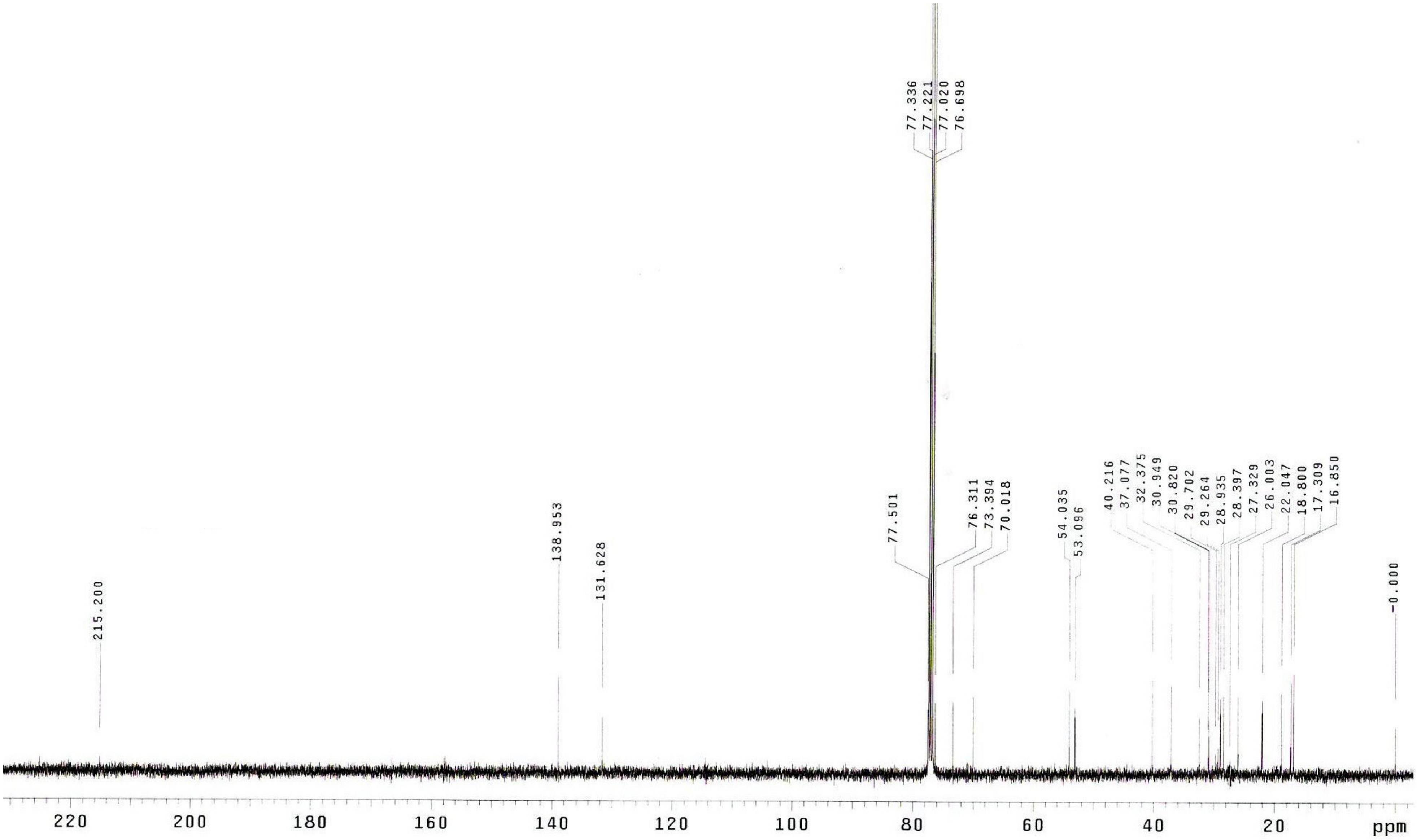

S4-2. ^{13}C NMR spectrum (400 MHz) of compound **4** in $CDCl_3$.

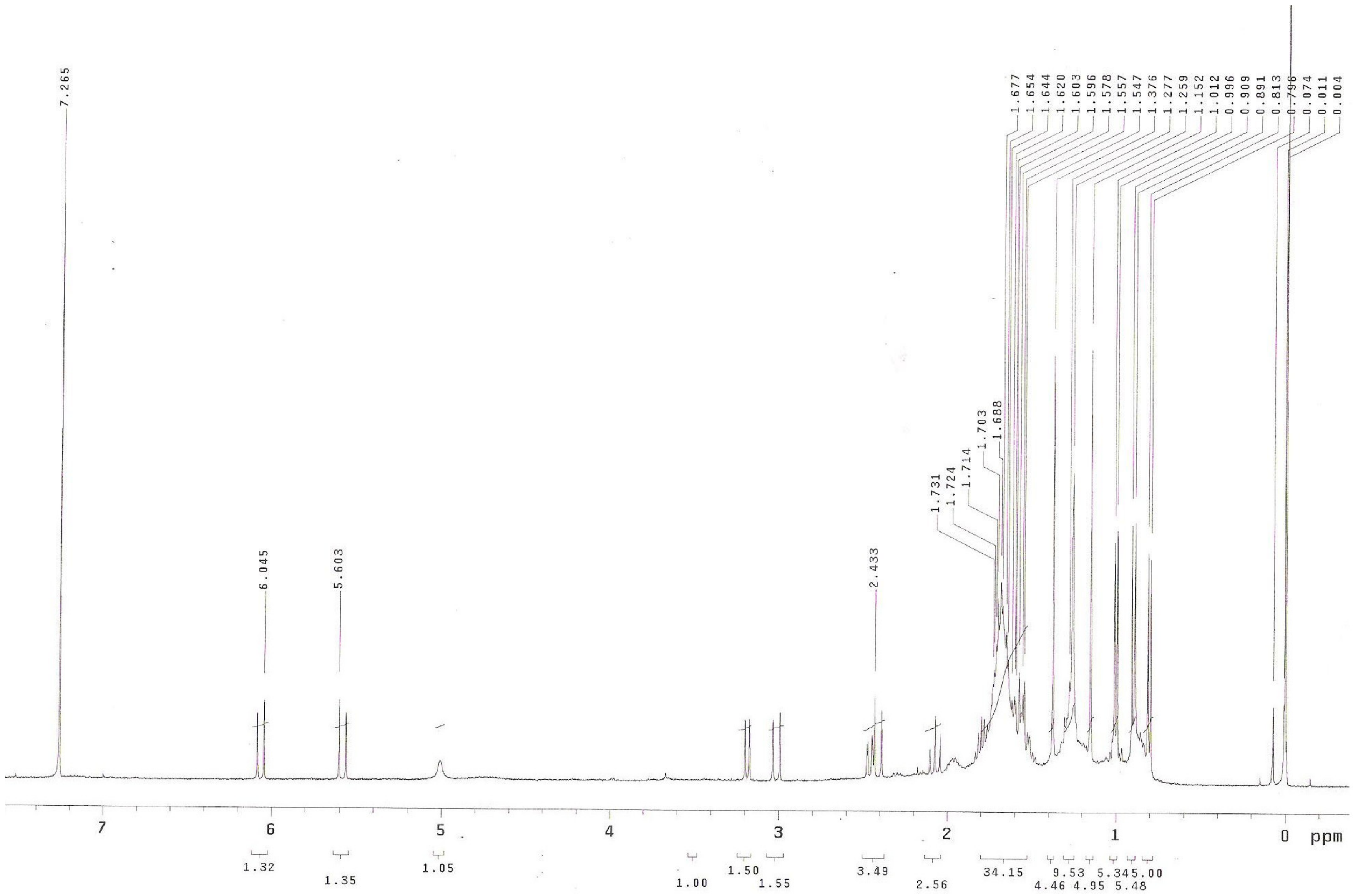

S5-1. ^{1}H NMR spectrum (400 MHz) of compound **5** in $CDCl_3$.

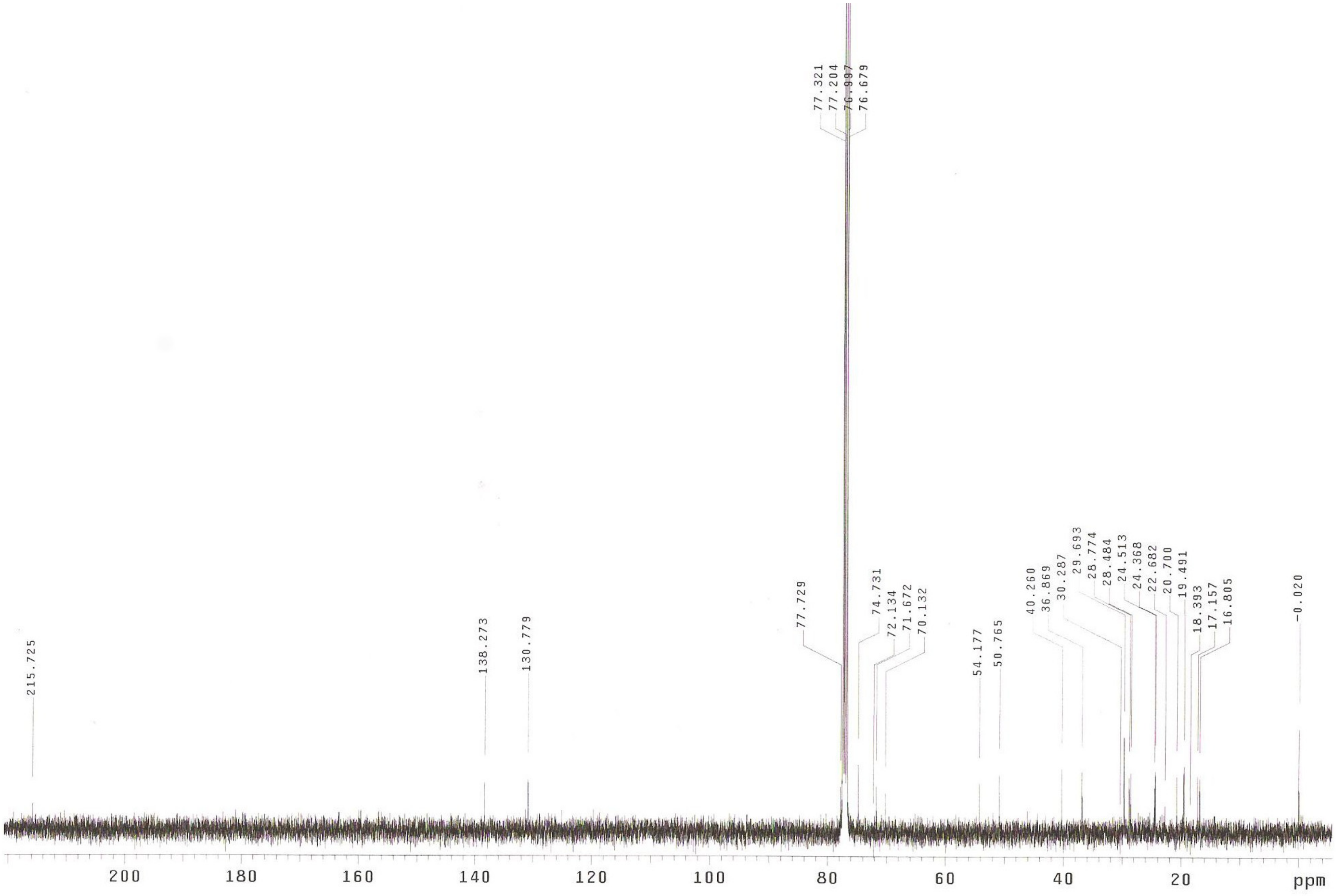

S5-2. ^{13}C NMR spectrum (400 MHz) of compound **5** in $CDCl_3$.

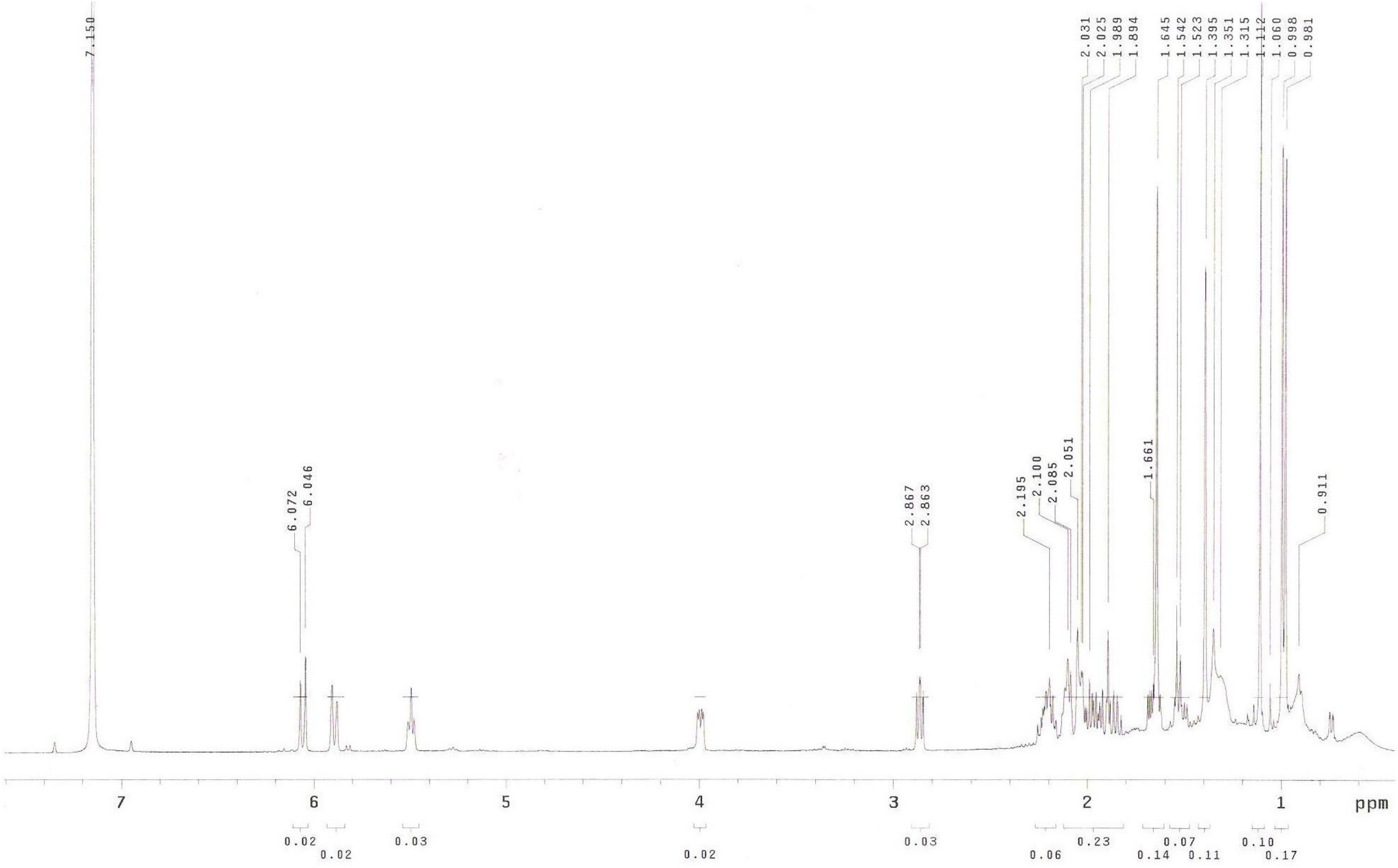

S6-1. ^{1}H NMR spectrum (400 MHz) of compound **6** in C_6D_6.

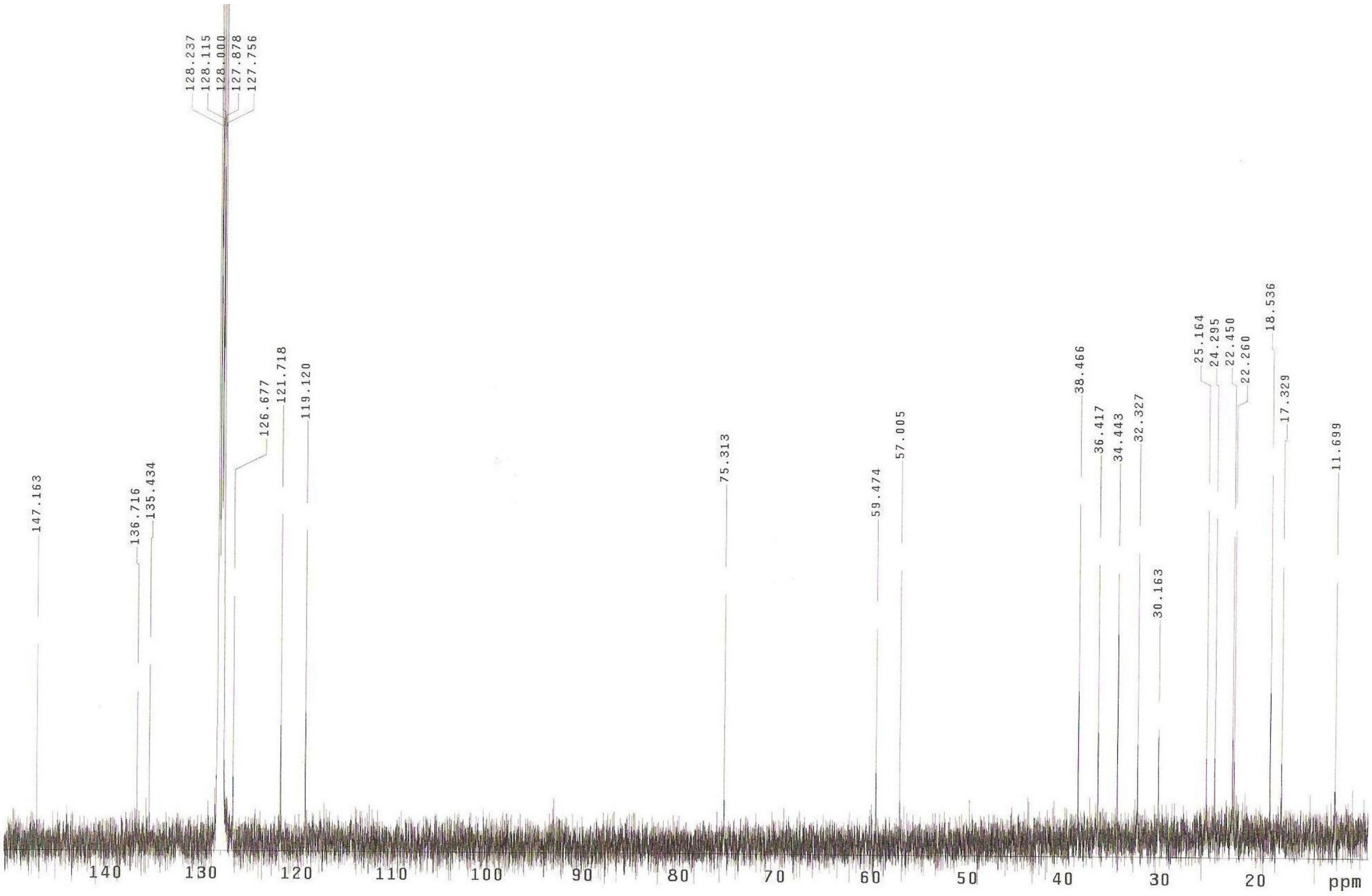

S6-2. ^{13}C NMR spectrum (400 MHz) of compound **6** in C_6D_6.

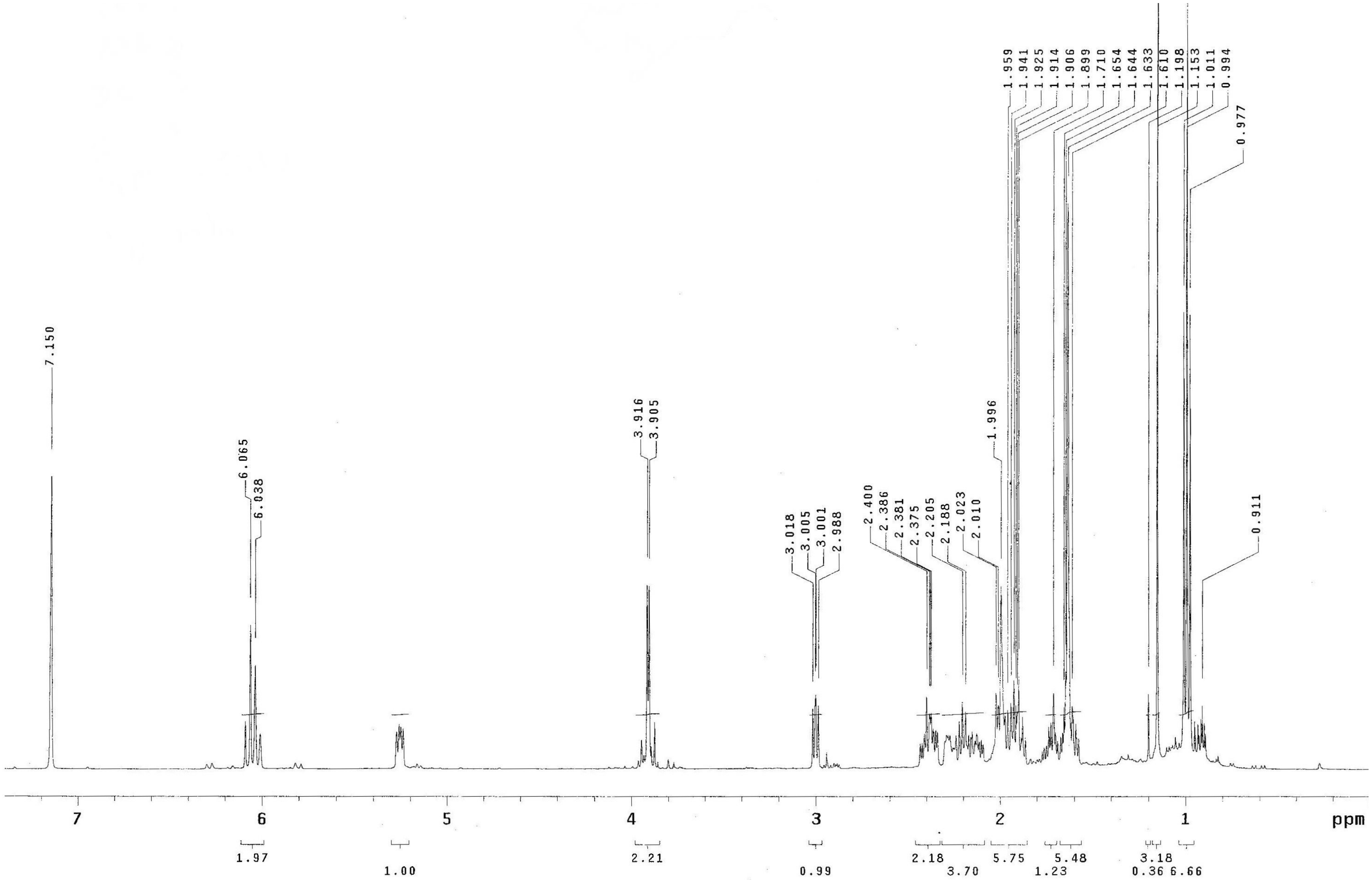

S7-1. ^{1}H NMR spectrum (400 MHz) of compound **7** in C_6D_6.

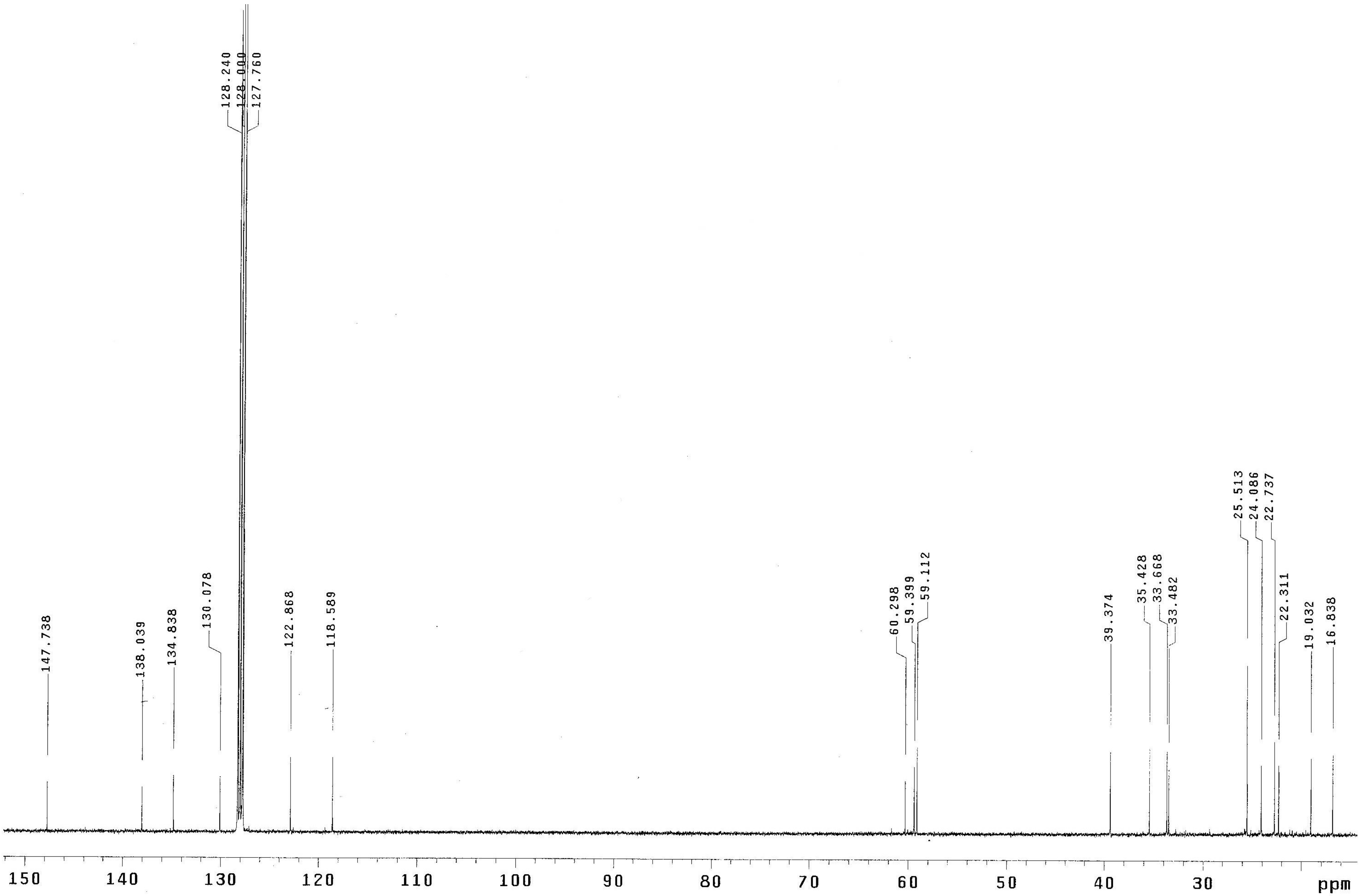

S7-2. ^{13}C NMR spectrum (400 MHz) of compound **7** in C_6D_6.

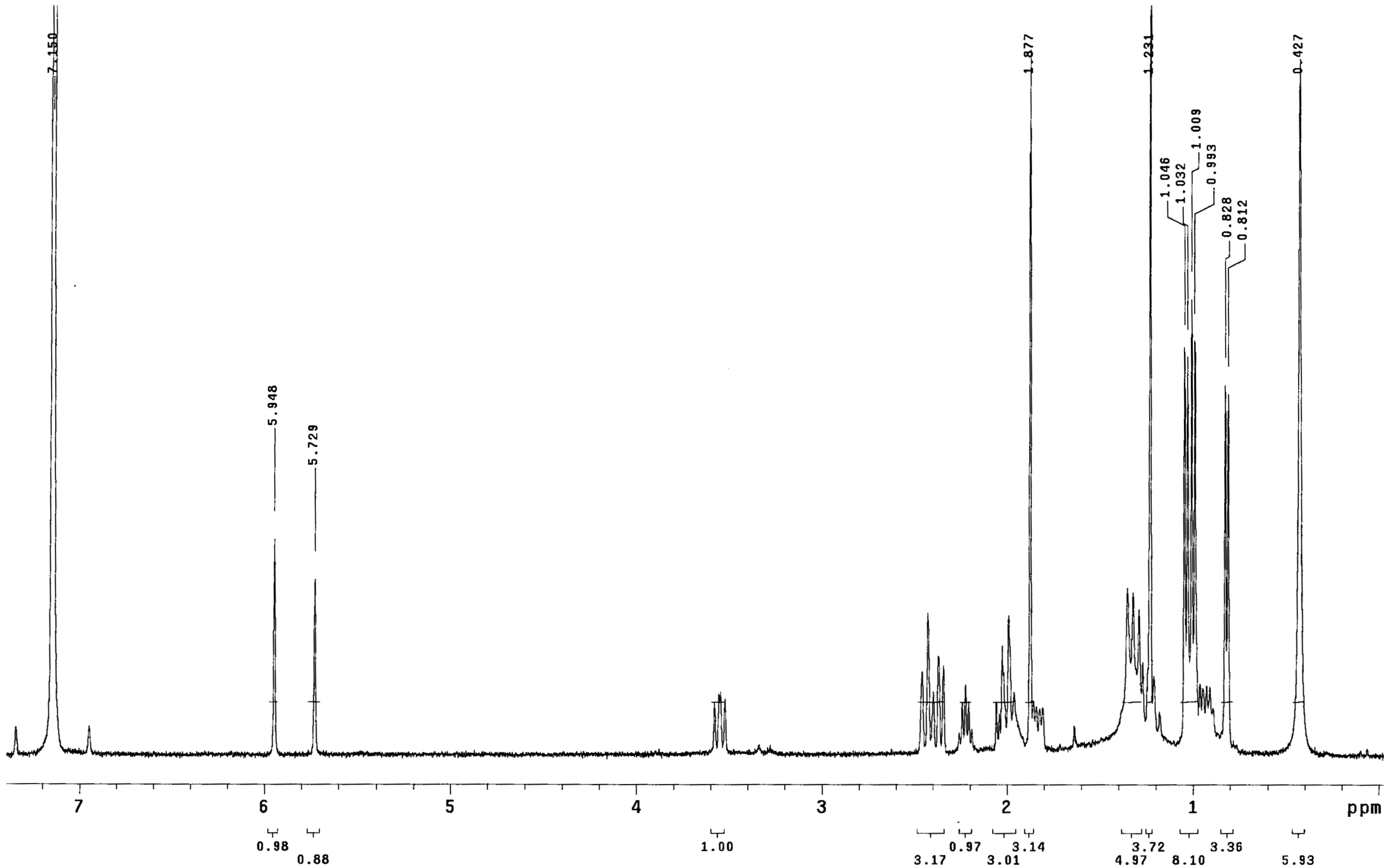

S8-1. ^{1}H NMR spectrum (400 MHz) of compound **8** in C_6D_6.

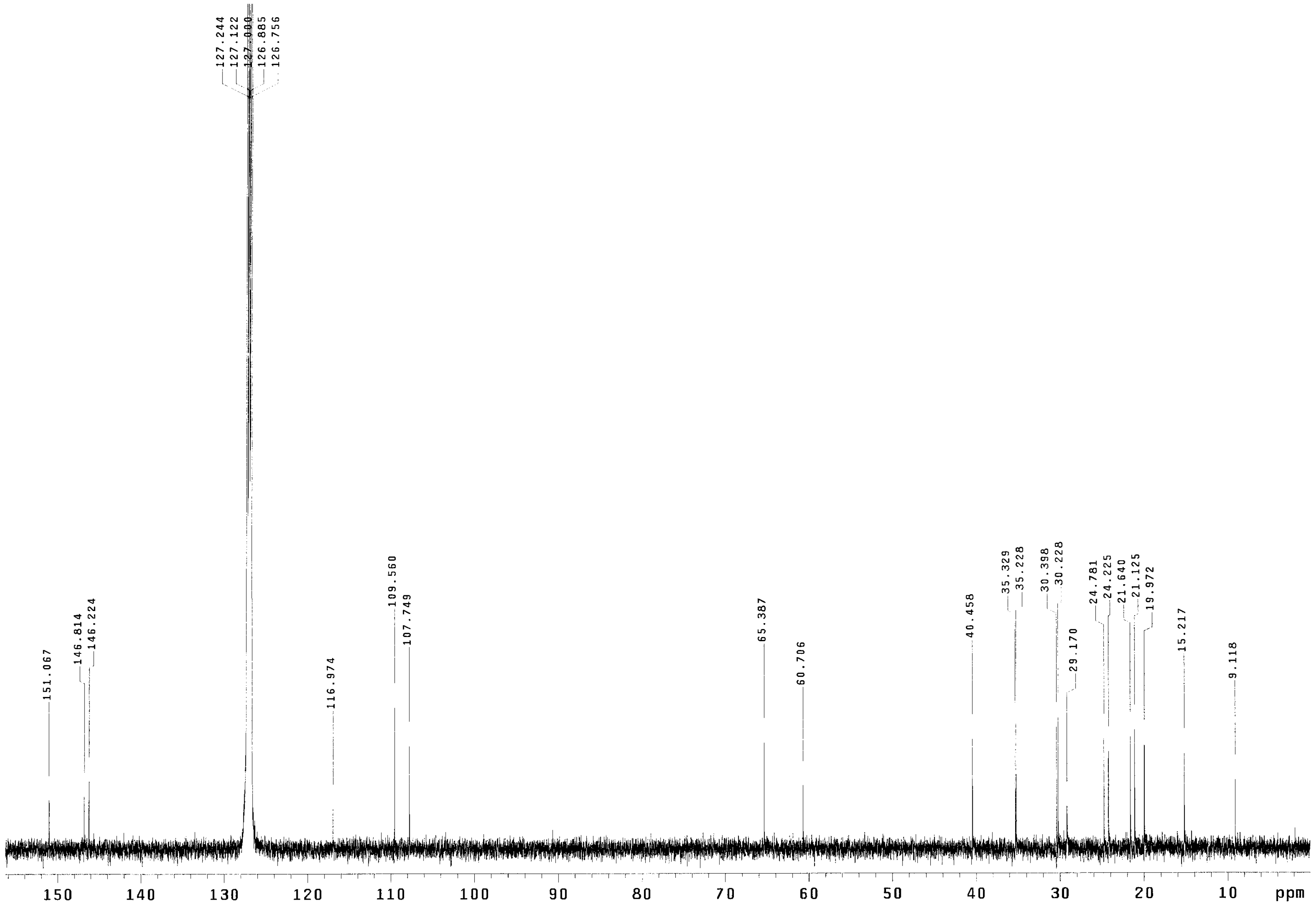

S8-2. ^{13}C NMR spectrum (400 MHz) of compound **8** in C_6D_6.
